# Expression of progerin enhances disease-related endpoints in a tau seeding reporter cell system

**DOI:** 10.1007/s11357-025-01737-z

**Published:** 2025-07-15

**Authors:** Zhuang Zhuang Han, Sang-Gyun Kang, Erik Gomez-Cardona, Serene Wohlgemuth, Klinton Shmeit, Luis Arce, Jiri G. Safar, Olivier Julien, David Westaway

**Affiliations:** 1 Department of Biochemistry, University of Alberta, 474 Medical Sciences Building, Edmonton, AB T6G 2H7 Canada; 2 Centre for Prions and Protein Folding Diseases, 204 Brain and Aging Research Building, University of Alberta, Edmonton, AB T6G 2M8 Canada; 3 Department of Pathology, Institute of Pathology Building, Case Western Reserve University, Rm 406, 2085 Adelbert Road, Cleveland, OH 44106-4907 USA; 4 Present Address: Cambridge Institute for Medical Research, Keith Peters Building, Biomedical Campus, Hills Rd, Cambridge, CB2 0XY UK

**Keywords:** Aging, Tau protein, Tauopathy, Aggregation, Lamin, Nuclear lamina

## Abstract

Sporadic Alzheimer’s disease and some forms of frontotemporal lobar degeneration (FTLD-tau) are neurological disorders of later life where cognitive deficits follow from the progressive accumulation of microtubule-associated tau protein. Disease-related tau accumulation is marked by altered subcellular distribution and rearrangement of this natively unstructured protein into alternative conformational forms, including highly organized fibrillar assemblies. With a partial analogy to effects seen in prion diseases, pathological tau conformers have a templating activity called seeding that may be measured in cellular and cell-free systems. Moreover, cellular systems and disease models can recapitulate “strain effects” wherein the same tau amino acid sequence can adopt markedly different conformations. Here we analyzed FTLD-tau conformers in cellular reporter systems expressing a pro-aging mutant form of the lamin A protein termed “progerin.” Measured versus the baseline performance of a reporter system based on HEK293 cells, the addition of tau burden or progerin expression produced only mild changes in proteomic analyses or morphology, whereas application of both stressors produced a notable shift in ER stress and homeostasis, including increased levels of DNAJC10 and DNAJA2. The phenotypic effects scored here appear unrelated to the generation of new tau strains or to the type of strain input, insofar as progerin-expressing cells were more responsive to tau seeding by diverse brain samples containing different populations of tau conformers. Thus, premature aging and disease-associated tau conformers can exhibit an additive relationship in a model system.

**Supplementary Information:**

The online version contains supplementary material available at 10.1007/s11357-025-01737-z.

## Introduction

Late-onset tauopathies, such as sporadic Alzheimer’s disease and other forms of senile dementia, represent a large family of age-related neurological disorders that are predominantly driven by pathological changes of tau. As human life expectancy continues to increase [1], these ailments have become a significant socio-economical challenge worldwide [2, 3]. Despite the urgent need of finding efficacious treatments, there are currently limited numbers of effective, disease-modifying interventions available, such as temporary symptomatic relief (e.g., cholinesterase inhibitors [4], serotonin reuptake inhibitors [5]). Slow progress in therapeutic development has been attributed, at least in part, to a lack of in vitro and in vivo models that accurately recapitulate an aged cellular environment [6]. In the field of cellular aging and neurodegeneration, approaches to study the effects of aging include the following (reviewed by [7]): first, the use of natural genetic variants that occur in animal lines that display phenotypes of premature aging— these would include animals harboring the polygenic SAMP8 trait or being homozygous for null alleles of the Klotho gene [8, 9]; second, the use of low expression levels of disease-related proteins (e.g., tau with pathogenic mutations) to attain pathologic changes at the end of the animals’ natural life span [10]; and third, the use of parabiosis to “age” young animals by infusing blood from older counterparts [11].

Among the genetic approaches, the use of mutant lamin A in the Hutchinson-Gilford progeria syndrome (HGPS) had attracted some attention in the context of aging-related neurodegeneration, due to its potential to circumvent the fixed timeline of chronological aging [12–14]. *LMNA* encodes for a nuclear envelope protein lamin A, which forms the nuclear lamina along with lamin B and lamin C [15]. In HGPS, a premature aging disorder, a mutation of *LMNA* activates a cryptic splicing site and leads to the production of a truncated protein (LMNA-Δ50, progerin) [16]. Once synthesized, progerin retains a farnesyl group at its C-terminus and thus becomes immobilized in the nuclear lamina. Stable association of progerin with the nuclear lamina has several sequelae, including thickened lamina, changes of the mechanical properties of the nuclei [17], alterations of chromatin architecture and genome stability [18, 19], and disruption of nucleocytoplasmic transport [20, 21], which overlap with the pathological phenotypes in the Alzheimer’s disease model [22]. Although the role of LMNA in the central nervous system remains controversial [23], multiple lines of evidence suggest that investigating its function in age-related neurological disorders is worthwhile. First, lamin A—normally absent in neurons—is reported to emerge during the transition from normal aging to Alzheimer’s disease (AD) neurons, where it helps counteract the effects of cell cycle re-entry and nuclear tau exit, thereby supporting neuronal survival [24]. Second, the expression of progerin in neurons promotes tau phosphorylation, amyloid plaque formation, and an increased Aβ42/Aβ40 ratio, all hallmark features of AD pathology [25].

Here, we utilized progerin expression as an avenue to mimic an aged environment in established tau aggregation reporter cells based upon a C-terminal tau construct containing four microtubule binding domains and a YFP fluorescent reporter moiety [26]. In earlier studies, disease associated forms of tau from human brain introduced into these reporter cells induced fluorescent puncta, some of which were localized to the nucleus. Notably, there are literatures describing tau in the nucleus [27, 28], including the deformation of nuclear membranes in the case of FTLD-MAPT mutations [29]. In experiments described below, expression of progerin in the absence of induced tau aggregates induced DNA damage and minor nuclear irregularities in the HEK cell system. Importantly, although progerin did not result in spontaneous formation of multimeric tau assemblies, cells stably expressing progerin consistently associated with a higher tau transduction efficiency when exposed to pathogenic tau. This progerin-induced sensitization for tau aggregation appeared to be a consequence downstream of tau seed uptake. Proteomic analyses of cells stably expressing progerin revealed a number of putative progerin-modulated regulators of tau aggregation in progeric cells, either with or without tau inclusions. Notably, several protein aggregation-related biological processes, such as autophagy and response to endoplasmic reticulum stress were emphasized in pathway analysis. Taken together, our results indicated that progerin expression enhances tau seeding when cultured cells are exposed to pathogenic tau species and such an effect is likely mediated by disruption of cellular protein degradation mechanisms.

## Methods

### DNA manipulations

c-Myc-progerin was constructed by fusing the c-Myc coding sequence (GAACAAAACTCATCTCAGAAGAGGATCTG) to the 5′ end of the progerin cDNA (Addgene, 17,663) via PCR reactions. The construct was subcloned into the mammalian expression vector pBudCE4 by using *Not*I and *Bgl*II restriction enzymes and verified by sequencing.

### Cell culture and transfection

Human embryonic kidney 293 cells that stably express tauRD-YFP fusion protein (HEK293 tauRD-YFP) were obtained from Dr. Marc Diamond (UT Southwestern, Dallas, TX) [26, 30]. The Edmonton Strain 1 (ES1) cell line was established in the Westaway lab by subcloning a tau inclusion-positive cell line from HEK293 tauRD-YFP cells that were infected with transgenic mouse brain-derived pathogenic tau [31]. The cells were grown in complete media: Dulbecco’s Modified Eagle Medium (Gibco™) containing 10% Fetal Bovine Serum (FBS, Gibco™) and 1% penicillin/streptomycin (Gibco™). Cells were cultured and passaged at 37 °C, 5% CO_2_, in a humidified incubator. 1 × sterilized PBS was used for washing cells prior to trypsinization with 0.25% trypsin–EDTA (Gibco™).

HEK293 tauRD-YFP and ES1 cells were passed with complete media until reaching 70% confluency. Five micrograms of each construct was mixed gently in a solution of Lipofectamine 3000 (Invitrogen) and OPTI-MEM media (Gibco™) at room temperature and incubated for 20 min prior to transfection. The DNA-lipofectamine mixture was then added to cells and incubated at 37 °C with 5% CO2 for 4 h in a humidified incubator. OPTI-MEM was then removed and replaced with DMEM containing 10% FBS. Transiently transfected cells were then cultured with DMEM (10% FBS) in the presence of 20 µg/mL zeocin (Invitrogen). Individual colonies were selected with cloning discs, expanded to reach confluency, and make derivative cell lines. In the case of progeric HEK293 tauRD-YFP clones (PH2 and PH3), we performed an additional step using limiting dilution to prepare single cell clones.

### Confocal microscopy

Eighteen-millimeter coverslips were coated with poly-D-lysine (5 µg/mL) and laminin (0.5 µg/mL) for 1 h and rinsed with 1 × PBS. Cells were grown for 24 h on double-coated coverslips. Media was removed and cells were rinsed with 1 × PBS for 5 min prior to fixation with 4% paraformaldehyde (PFA) for 15 min, and permeabilization with 0.25% Triton-X for 15 min. Coverslips were blocked with 1% BSA-PBST for 30 min and incubated with primary antibodies, anti-γH2AX (1:500, Novus Biologicals, NB100-384) and anti-c-Myc (1:500, GenScript, A00704), at 4 °C overnight. Primary antibodies were aspirated and replaced with 1 × PBS for a 3 × 5 min rinse. Cells were then incubated with Alexa Fluor 647-conjugated secondary antibody (Invitrogen, 1:400 diluted in 1% BSA-PBST) at 22 °C, in the dark, for 1 h. Cells were washed with 1 × PBS before being mounted with Prolong Gold Antifade Mountant with DAPI (Invitrogen), sealed with nail polish, and stored at 4 °C before confocal analysis. For confocal microscopy, a Zeiss LSM 700 laser scanning confocal microscope was coupled to a X-Cite mini + compact LED illumination system.

### Image analysis for nuclear dysmorphology by Aiforia

On day 1, 96-well plates (Greiner Bio-One) were double-coated with poly-D-lysine (5 µg/mL) and laminin (0.5 µg/mL) for 1 h and rinsed with 1 × PBS. Six cell lines (NPH, PH2, PH3, NPE, PE7, and PE9) were seeded in a 96-well plate with a density of 10,000 cells/well before being incubated at 37 °C with 5% CO2 for 24 h in a humidified incubator. On day 2, cells were fixed with 4% PFA and 0.25% Triton-X. The plate was then blocked with 1% BSA-PBST for 30 min and incubated with primary anti-lamin B1 antibody (Abcam, ab16048) at 4 °C overnight. Cells were then incubated with Alexa Fluor 594-conjugated secondary antibody (Invitrogen, A11037, 1:400 diluted in 1% BSA-PBST) at 22 °C, in the dark, for 1 h. Cells were washed with 1 × PBS before being stained with 10 µg/mL Hoechst stain dissolved in 1 × PBS. Cells were imaged with DAPI and 594 nm channels at 60 × resolution by the MetaXpress XLS High Content Analysis System (Molecular Devices).

Cell images with lamin B1 staining were subsequently analyzed by Aiforia (https://www.aiforia.com/) with the following steps: first, 5–10 cells/image were manually annotated over 30 cell images. Two categories were used for nuclear morphology classification: “negative” representing cells with normal-looking nuclei and “positive” representing cells with aberrant nuclear morphology, including abnormal shape, nuclear blebs, nuclear envelope invagination, and micronuclei, as reviewed by [32]. Second, the model was established with manually annotated cells with 200 iterations, 2000 iterations, and 20,000 iterations. The accuracy of the model was verified by comparing computer-generated results to manual quantitation of five cell images (~ 40 cells/image). Once its accuracy was confirmed, the model (i.e., computer-based analysis pipeline) was then applied to all 480 images for quantitative nuclear morphology measurement (20,000 iterations).

### Semi-denaturing detergent agarose gel electrophoresis

SDD-AGE was performed as previously described with minor modifications [26]. Briefly, cell pellets lysed in 1 × RIPA buffer were clarified by sequential centrifugations (500 × g, 1000 × g). Low-SDS 1% agarose gels were prepared by dissolving agarose in 1 × TAE buffer (40 mM Tris, 20 mM acetate, 1 mM EDTA, pH 8.6) with 0.02% SDS. For each sample, 5 µg of clarified cell lysate was incubated with sample buffer (1 × TAE, 0.02% SDS, 5% glycerol, 0.05% bromophenol blue) at 22 °C for 5 min. Protein was capillary transferred to Immobilin P (Millipore) with the gel running buffer (1 × TAE, 0.02% SDS) for 2 h. Membranes were probed for tau with mouse polyclonal anti-tau ET3 (1:250, generously provided by the Davis Laboratory) and counter-probed with goat anti-mouse HRP (1:10,000, Invitrogen).

### Analyses of protein expression with capillary western immunoassay

Cell lysates were normalized to 2 µg/µL with the bicinchoninic acid (BCA) assay. 0.1X Sample Diluent (ProteinSimple) was used to dilute cell lysate samples. Fluorescent 5X Master Mix (ProteinSimple) was used to denature protein samples at 95 °C for 5 min. Primary antibodies—RD4 (1:250, MilliporeSigma, 05–804), anti-β-tubulin (1:400, Novus Biologicals, NB600-936), anti-c-Myc (1:500, GenScript, A00704) and anti-GAPDH (1:25, Novus Biologicals, NB300-221) were diluted with Antibody Diluent 2 (ProteinSimple). Secondary antibodies (anti-mouse or anti-rabbit, ProteinSimple) were used undiluted according to the instructions from ProteinSimple. 12–230 kDa separation modules were used for all analyses. The Compass software (ProteinSimple) was used for generating the “lane view” from chromatographs and calculating “area under the curve” for quantitative comparison.

### Tau protein transduction in HEK293 tauRD-YFP cells

On day 1, 96-well plates (Greiner Bio-One) were double-coated with poly-D-lysine (5 µg/mL) and laminin (0.5 µg/mL) for 1 h and rinsed with 1 × PBS. HEK293 tauRD-YFP cells were seeded in a 96-well plate with a density of 5000 cells/well before incubated at 37 °C with 5% CO2 for 24 h in a humidified incubator. On day 2, tau extracts (brain-derived or cell lysate), Lipofectamine 3000 (Invitrogen) and OPTI-MEM media (Gibco™) were mixed at room temperature and incubated for 20 min prior to transduction. The working ratios between tau extracts and Lipofectamine 3000 were experimentally determined by titration (Fig. S1). Total tau concentration in each brain homogenate was measured by conformation-dependent immunoassay [33] and normalized to 8000 ng/mL. Lysates from the ES1 cell line were adjusted depending upon total protein concentration. Complete media was aspirated and replaced with 100 µL of warm OPTI-MEM media per well. Ten microliters of pre-mixed liposome-tau complex was added to each well. After a 4-h incubation, 100 µL of warm complete media was added to each well and cells were incubated at 37 °C with 5% CO2 for 24 h in a humidified incubator. On day 3, cells were incubated at 30 °C with 5% CO2 for 24 h in a humidified incubator to prevent the cells from over-proliferating. On day 4, cell culture media was aspirated, and cells were fixed with 4% PFA for 15 min. One hundred microliters of mounting medium (70% glycerol, 500 µg/mL p-phenylenediamine and 10 µg/mL Hoechst stain) was then added to each well. Cells were imaged with DAPI and GFP channels at 10 × resolution and quantified by the MetaXpress XLS High Content Analysis System (Molecular Devices).

### Limited proteolysis

ES1 cell pellets were frozen at − 80 °C for storage. On the day of cell lysis, cell pellets were thawed on ice and lysed in 1 × PBS with 0.05% Triton X-100 and a complete mini protease inhibitor tablet (Roche). Sequential 5-min centrifugations were performed at 500 × g and 1000 × g to clarify the cell lysate at room temperature. A BCA assay with BSA standard curve was then performed and protein concentrations were adjusted to 1.7 µg/µL with addition of lysis buffer.

Lyophilized pronase E (Roche) was re-suspended in 1 × PBS to a final concentration of 1 µg/µL and aliquots were stored at − 80 °C. Lyophilized thermolysin (Sigma) was re-suspended in storage buffer (50% glycerol, 0.1 M sodium acetate, 0.5 mM calcium acetate, pH 7.5) to a final concentration of 1 µg/µL and aliquots were stored at − 20 °C.

Titration experiments were performed to determine the working ratios between proteases and cell lysate. The cell lysates (3.5 µg protein) were digested with 500 ng of pronase E at 37 °C for 1 h, or 50 ng of thermolysin at 65 °C for 30 min, or 250 ng of proteinase K at 37 °C for 30 min. Pronase E was quenched with protease inhibitors (Roche) and SDS-PAGE loading buffer; thermolysin was quenched with 0.5 M EDTA and SDS-PAGE loading buffer; proteinase K was quenched with SDS-PAGE loading buffer. The undigested tau fragments in each enzymatic reaction were determined by western blot analysis using anti-tau mAb ET3.

### Mass spectrometry sample preparation and analysis

To explore progerin-induced changes in cellular proteomics, six different cell lines (non-progeric HEK293 “NPH,” non-progeric ES1 “NPE,” progeric HEK293 clone 2 “PH2,” progeric HEK293 clone 3 “PH3,” progeric ES1 clone 7 “PE7,” and progeric ES1 clone 9 “PE9”) were analyzed by liquid chromatography with tandem mass spectrometry (LC–MS/MS) with a label-free quantitation approach. Approximately 5 million cells per sample were lysed in a urea-based lysis buffer (8 M urea, 100 mM Tris pH 8.5, 5 mM EDTA, 1 mM AEBSF, 1 mM PMSF, and 4 mM IAM). Total protein concentration of each sample was determined by BCA assay (ThermoFisher Scientific). Each cell line was analyzed in triplicates. Digestion of 100 µg of total protein per sample was performed using the Cytiva Sera-Mag™ Carboxylate modified Magnetic Beads in a KingFisher Duo Prime Purification System. Samples and reagents were loaded into 96-well KingFisher plates. An automated on-bead digestion protocol was created using the BindIt software (v4.0.0.45). The protocol included bead collection and wash in 80% ethanol, followed by protein capture, bead washes with 80% ethanol, reduction and alkylation (10 mM DTT and 25 mM IAM), wash with 100 mM NH_4_HCO_3,_ followed by on-bead trypsin digestion. Peptides were recovered from the plate and desalted using C18 Ziptips (ThermoFisher Scientific).

For mass spectrometry analysis, samples were dissolved in H_2_O with 4% (v/v) ACN and 0.1% (v/v) formic acid and separated using a nanoflow-HPLC (Thermo Scientific EASY-nLC 1200 System) coupled to an Orbitrap Fusion Lumos Tribrid Mass Spectrometer (Thermo Fisher Scientific). Peptides were loaded into a trap column (5 µm, 100 Å, 100 µm × 2 cm, Acclaim PepMap 100 nanoViper C18; Thermo Fisher Scientific) and resolved with an analytical column (2 µm, 100 Å, 50 µm × 15 cm, PepMap RSLC C18; Thermo Fisher Scientific). Reverse phase separation of the peptides was done using a 120 min linear gradient from 3.85 to 36.8% acetonitrile in 0.1% formic acid.

Label-free quantitation of the proteins was performed in the software ProteinDiscoverer (v2.4) using the LFQ processing workflow against the Uniprot human proteome data (2020, 42,285 sequences) with the Myc-tagged progerin sequence added. The search was performed with a maximum false discovery rate of 1% for peptides. Trypsin was selected as a digestion enzyme with a maximum of 2 missed tryptic cleavages. Precursor mass tolerance was set at 15 ppm with a fragment mass tolerance of 0.8 Da. Carbamidomethylation (C) was added as a fixed modification and, deamidation at N/Q and M oxidation were set as variable modifications. Only proteins identified with two or more peptides and observed in two or three replicates were considered for comparison among groups. We used the Consensus workflow: Comprehensive Enhanced Annotation for Label-free quantification and precursor quantification from Proteome Discoverer, which includes a *t*-test (background based) with a protein ratio calculation that uses the median of all possible pairwise peptide ratios calculated between replicates to reduce outliers and missing values. Lists of significantly upregulated and downregulated proteins were generated with: *p*-value < 0.01 and fold change > 1.5.

### Gene set bioinformatics enrichment analysis

Gene Ontology (GO) term enrichment analyses were performed for the significantly modulated proteins using the MetaScape database (http://metascape.org/) [34]. The major GO terms of the DE genes in biological processes were illustrated. The parameter settings were as follows: (1) minimum overlap is 3; (2) the cut-off *p*-value = 0.01; (3) the minimum enrichment score = 1.5.

### Heatmap generation and hierarchical clustering

Grouped scaled abundance for every significantly increased or decreased protein for the comparisons PH2&PH3 vs NPH and PE7&PE9 vs NPE were extracted from the ProteomeDiscoverer protein report. Zero filing using a local minimum approach was applied. Heatmaps for the abundance distribution of these proteins were generated using Morpheus (https://software.broadinstitute.org/morpheus). Hierarchical clustering using the Pearson correlation metrics with average linkage was selected to create clusters with similar protein abundances, and a cutoff of 6 clusters was selected.

### Statistical analysis

The number of independent experiments or biological replicates of compared groups were *n* = 3 for each observation. Statistical analysis for the quantitative data including capillary western immunoassay and tau transduction efficiency analysis was performed using one-way ANOVA. 

## Results

### Generation of progeric HEK293 tauRD-YFP reporter cell lines

**Fig. 1:**
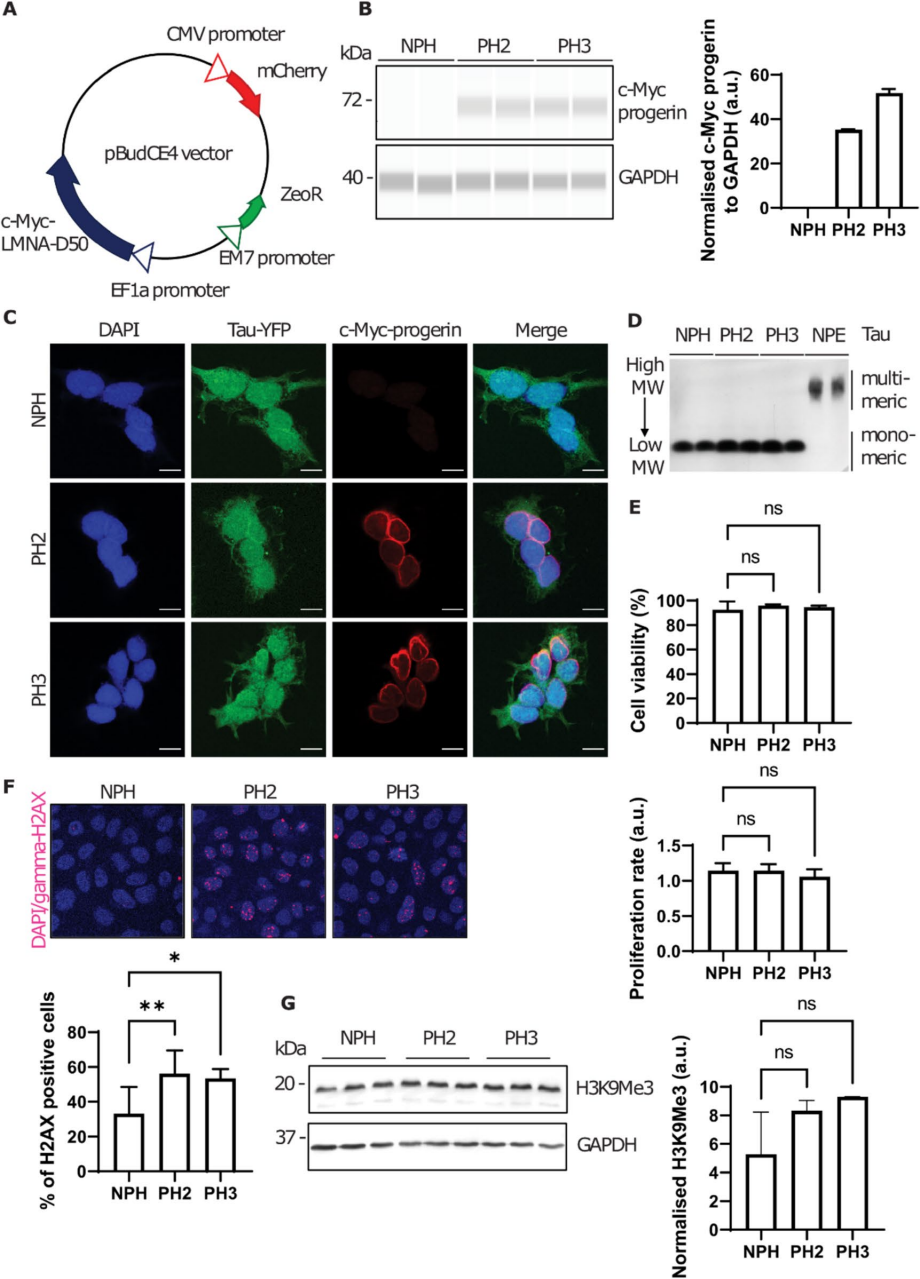
Generation of progeric HEK293 tauRD-YFP monoclones. **A** Schematic diagram of the pBudCE4 vector used to generate the progeric tau reporter cell line. **B** Capillary western immunoassay analysis displayed assimilation of the LMNA-Δ50 transgene construct in two independent HEK progeric stable clones. “Lane view” of the capillary western immunoassay was shown and the average signal intensity for c-Myc-progerin and GAPDH was represented in the bar graph. c-Myc tagged progerin was probed by anti-c-Myc antibody. Progeric clone 2 and clone 3 (PH2 and PH3), along with non-progeric control cells (NPH), were used for further experiments. Error bar = S.D. **C** Immunocytochemistry analysis displayed nuclear membrane-bound c-Myc-progerin. c-Myc tagged progerin was probed by anti-c-Myc antibody (primary) and Alexa Flour 647 (secondary). Scale bar = 10 µm. **D** SDD-AGE analysis demonstrated that PH2 and PH3 did not spontaneously form multimeric tau species, in comparison to the non-progeric ES1 cells (NPE) that were chronically infected with pathogenic tau. NPE Tau was probed by anti-tau ET3 antibody. **E** Trypan blue exclusion test of cell viability showed similar measurement for progeric and non-progeric HEK293 TauRD-YFP clones. LDH activity assay showed a similar proliferation rate for progeric and non-progeric cells. **F** Immunocytochemistry analysis of phosphorylated histone γH2AX (DNA damage marker) in progeric and non-progeric clones. Scale bar = 20 µm. Percentage of γH2AX-positive nuclei was represented in the bar graph. *p < 0.05; **p < 0.01. **G** Western blot analysis of H3K9me3 (epigenetic modification of histone 3) in progeric cell lines. Average signal intensities of H3K9me3 bands were shown in the bar graph. Error bar = S.D

To determine the impact of stable expression of progerin in a tau aggregation reporter model, we transfected a previously established HEK293 tauRD-YFP reporter cell line [26, 30] with c-Myc tagged progerin expressed from a housekeeping gene promoter (EF-1α; Fig. 1A) and selected two independent monoclonal cell lines. These cell lines, “PH2” (progeric HEK293 clone 2) and “PH3” (progeric HEK293 clone 3), proved to stably express progerin at a similar level, with expression absent from untransfected, “NPH” (non-progeric HEK293) negative control cells (Fig. 1B). Nuclear localization of c-Myc tagged progerin was confirmed in PH2 and PH3 cells with immunocytochemistry (Fig. 1C). Additionally, a diffused signal from the tau-YFP fusion protein suggested that progerin expression did not per se result in spontaneous formation of tau aggregates. In agreement with this, separation of protein species by semi-denaturing detergent agarose gel electrophoresis (SDD-AGE), showed that PH2 and PH3 did not form high molecular weight multimeric tau species in the absence of external tau seeds (Fig. 1D). Trypan blue exclusion test of cell viability and LDH (lactate dehydrogenase) activity assay showed similar viability and proliferation rates for the PH2 and PH3 progeric cells and the NPH non-progeric HEK293 tauRD-YFP controls (Fig. 1E). Consistent with reports from other groups [12, 19, 35, 36], expression of progerin was correlated with an increased level of double-stranded DNA damage, as indicated by focal signals with an anti-phosphorylated histone γH2AX antibody (Fig. 1F). It is important to note that γH2AX accumulation does not necessarily correlate with cell death. For example, primary progeria fibroblasts exhibit elevated γH2AX levels without undergoing premature cell death; instead, they enter senescence earlier [37]. In our study, since HEK293 cells are immortalized, the senescence barrier is bypassed. Additionally, an elevated level of trimethylated histone H3, H3K9me, in the progeric cell lines (Fig. 1G), although not reaching statistical significance, indicated that progerin expression may alter the epigenome in these cells; this observation is consistent with observations made in low-passage HEK293 cell lines that express progerin or unprocessed prelamin A [38].

### Progerin expression increases seeding of HEK293 tauRD-YFP reporter cells

**Fig. 2:**
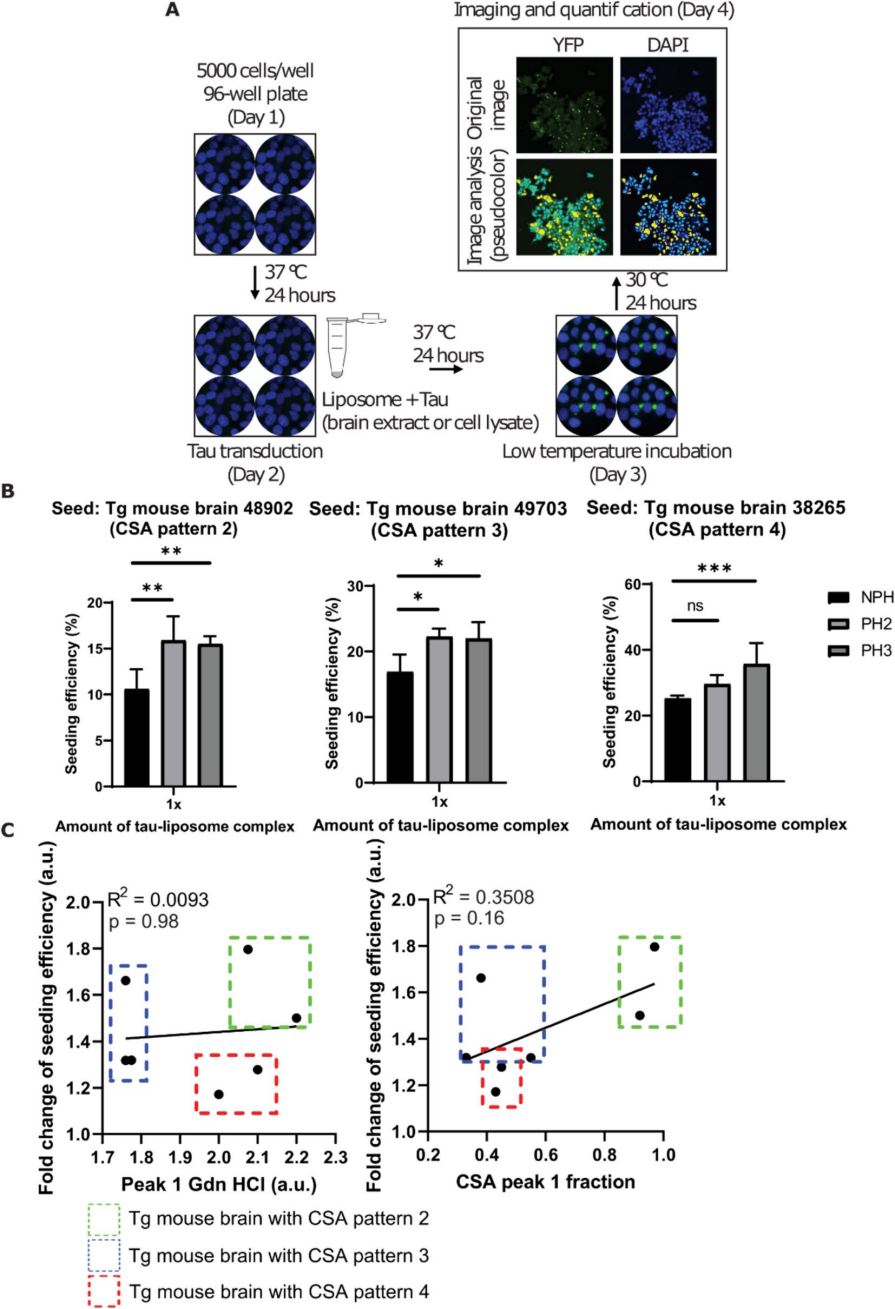
Progerin expression enhances the prion-like propagation of tau aggregation. **A** Schematic illustrating methods used to transduce brain-derived or cell lysate-derived tau extracts into HEK293 tauRD-YFP cells (progeric or non-progeric), followed by automated quantitation of fluorescence output. **B** Relative tau seeding efficiency into progeric versus parental cells. HEK293 cells (progeric and non-progeric) were transduced with preparations that contain pathogenic tau, including TgTau mouse brain extracts and ES1 cell lysate. The total amount of tau used per 96-well plate well ranged from 0.87 ng to 8.7 ng, depending on the sample (Fig. S1). The total amount of tau-lipofectamine mixture used for transduction was increased from a starting concentration 1 × to 2 × or 3x. The seeding efficiency of two progeric clones (light red = PH2 and dark red = PH3) was compared to that of non-progeric cells (blue = NPH). Error bars represent mean ± S.E. of *n* = 3 biological replicates. *p < 0.05, **p < 0.01, ***p < 0.001 (two-way ANOVA). **C** Pearson correlation analysis for progerin-induced increase of seeding efficiency in PH2 cell line and the chemical signature (peak 1 Gdn HCl and CSA peak 1 fraction) generated by the CSA assay. Seven Tg mouse brain extracts were used in the analysis (two brains with CSA pattern 2, three brains with CSA pattern 3 and two brains with CSA pattern 4)

To analyze the effect of progerin on intracellular tau aggregation, we seeded cells (progeric and parental controls) with brain extracts derived from TgTau^P301L^ transgenic mice expressing human 4R tau [39] or from lysates of cells bearing tau inclusions. Evaluation of the frequency of cells with tauRD-YFP inclusions was performed using high content fluorescence microscopy (Fig. 2A). The ratios between tau extract and lipofectamine reagent that were most effective in producing fluorescent foci were determined empirically in titration experiments (Fig. S1). Also, mixing experiments with different ratios of unseeded cells and ES1 cells chronically “infected” with pathogenic tau [40] showed an apparently linear relationship between ES1 cell input and the percentage of cells exhibiting fluorescent foci for this assay configuration (Fig. S2), suggesting that the fluorescent foci analysis pipeline is reliable.

Brain extracts from seven aged low-expresser tau transgenic mice (TgTau(P301L)23,027 line) previously profiled by a conformational stability assay (CSA) [33] were used as starting material for tau transduction experiments. Briefly, stepwise addition of the protein denaturant guanidine hydrochloride (Gdn HCl) in a CSA unfolds aggregated tau and exposes an epitope that is hidden under native conditions (i.e., in the absence of Gdn HCl). By probing this epitope with a europium-labeled antibody, a guanidine-titration stability profile can be generated to provide structural information of misfolded tau [33]. Among these seven brain samples, two were associated with a denaturation profile denoted CSA type 2, three were associated with a CSA type 3 profile, and a further two were associated with a CSA type 4 profile, noting that the midpoint of these profiles shifts to the right (i.e., to higher Gdn HCl concentrations) proceeding from CSA type 2 through to type 4 profiles. Since a homogenous population of conformers will generate a simple sigmoidal signal in response to unfolding and since these tau preparations exhibit complex unfolding signals in response to increasing Gdn HCl concentrations [33], it has been deduced that these brain-derived tau preparations (from either human FTLD-MAPT cases or a mouse FTLD-MAPT model) contain a mixture of conformers. In previous analyses, the mixture of conformer species in a human or animal brain has been referred to as an “ensemble.”

Seeding experiments performed with these brain-derived tau ensembles revealed that progeric HEK cells (PH2 and PH3) consistently associated with higher levels of seeding compared to the control non-progeric HEK parental cells (NPH) when treated with Tg mouse brain-derived tau preparations or with sarkosyl-insoluble tau sedimented from ES1 cells (Fig. 2B and **S3**). It is noteworthy that the total amount of tau-lipofectamine mixture did not seem to influence the relative elevation of seeding efficiency in the progeric cells versus the control non-progeric HEK parental cells (Fig. S3, 1x, 2x, and 3x).

To determine if the increased seeding efficiency in progeric cells favored specific strains of tau seeds (i.e., different types of tau conformer ensembles, as characterized by biophysical or biochemical methods), we assessed the fold change of seeding efficiency in progeric cells versus quantitative measurements of tau seeds that were generated by the CSA assay. Thus, we plotted “peak 1 Gdn HCl” or “CSA peak 1 fraction” (characteristic measurements of seven Tg mouse brain-derived tau preparations [33]) on the abscissa versus the progerin-induced fold change of seeding efficiency displayed on the ordinate axis (Fig. 2C and **S4**). However, we observed no statistically significant correlation between seeding efficiency and the chemical signature of pathogenic tau. Despite that CSA pattern 2 samples generally associated with a larger increase in seeding efficiency and CSA pattern 4 samples generally associated with a smaller increase in seeding efficiency, these data suggest that the increase of seeding efficiency observed in progeric cells is largely unrelated to the putative strain composition of the tau seeds, and in this regard represents a generalizable effect.

### Progerin expression does not affect tau seed uptake

To reveal the molecular mechanisms by which progerin expression led to an increase in the seeding efficiency in tau aggregation reporter cells, we considered the following explanations:

1. Progerin expression results in an altered expression level of tauRD-YFP reporter protein.
2. Progerin expression results in more efficient uptake of lipofectamine-packaged particles.
3. Progerin expression allows for direct entry of tau seeds into the cytoplasm without lipofectamine.
4. Progerin expression leads to a different conformation of pathogenic tau, which allows for more efficient templated conversion of tauRD-YFP fusion protein substrate into forms discerned as fluorescent foci.
5. Progerin expression leads to disruption of homeostasis in cultured cells, in particular degradation activities that dissassemble misfolded tauRD-YFP fusion proteins.

**Fig. 3:**
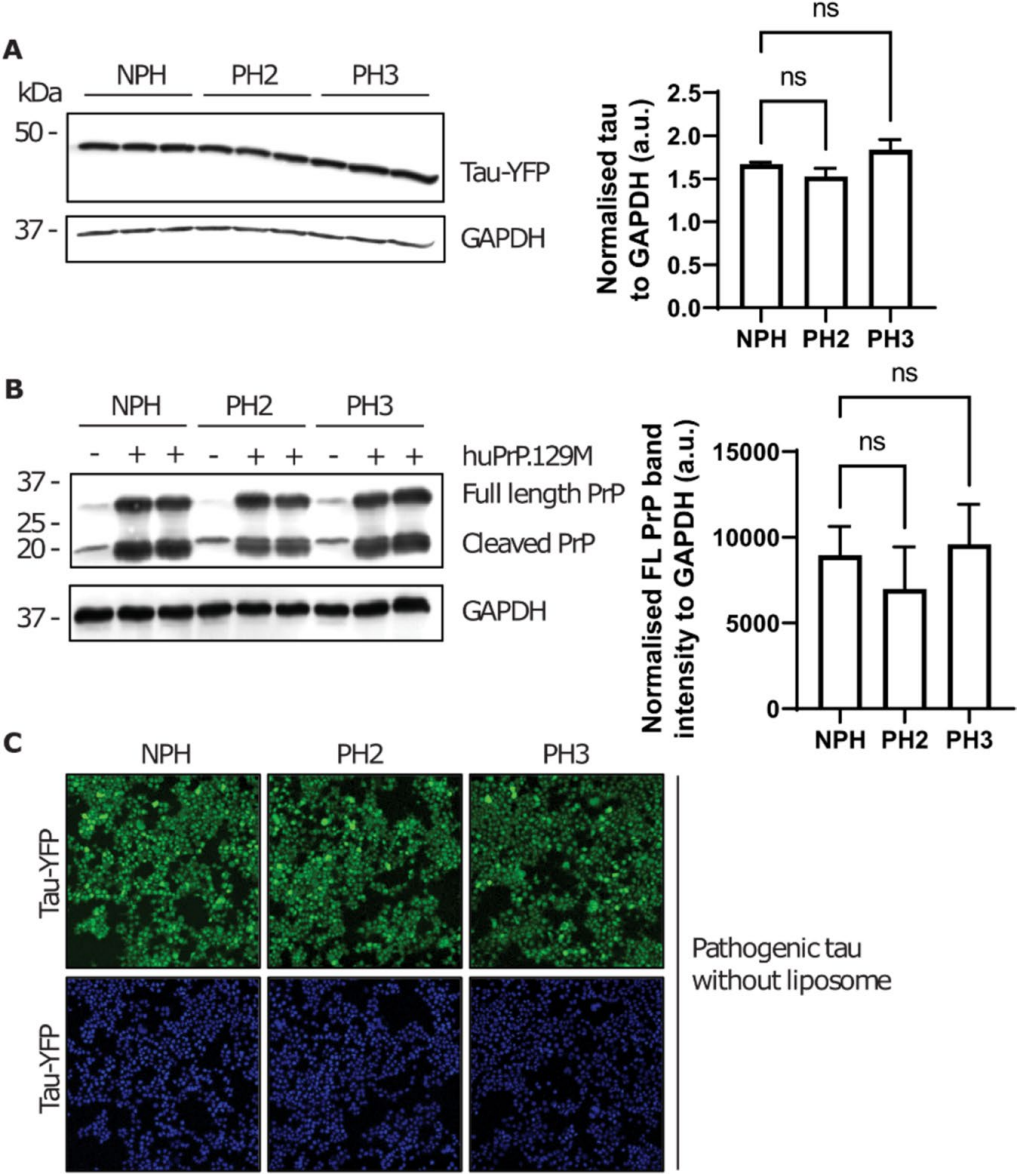
Progerin expression and pathogenic tau seed uptake. **A** Expression of progerin did not impact steady-state levels of the tauRD-YFP construct in HEK reporter cells, as quantified by immunoblot. **B** Western blot analysis displayed similar expression levels of human PrP in progeric and non-progeric cells after transient liposome-mediated transfection. The normalized signal intensity for full length PrP was represented in the bar graph. PrP was probed by Sha31 antibody. Error bar = S.E. **C** Sample image of progeric and non-progeric cells that were treated with a TgTau^P301L^ mouse brain-derived extract of pathogenic tau in the absence of lipofectamine. No intracellular puncta were detected under these conditions

Regarding the first option, we assessed whether or not progerin expression alters the level of our tau reporter fusion and excluded this interpretation (Fig. 3A). Regarding the second option, since tau seed uptake was dependent on the use of liposome in our procedures, we assessed if cells stably expressing progerin are more easily transfected with lipofectamine. However, when transfected with an internal control expression vector encoding human prion protein (huPrP.129 M), non-progeric and progeric cells had the same steady-state expression level of two abundant forms of PrP (full-length molecules and cleaved C-terminal fragments, “C1”, as measured after chemical deglycosylation; Fig. 3B). Regarding the third possibility, since it has been previously reported that certain tau seeds may enter the cells without liposomes [41], we tested whether or not progerin expression impacted the uptake of tau seeds in a liposome-independent manner. To this end, we found that progerin expression did not impact tau seed uptake. In fact, pathogenic tau seeds that were derived from TgTau mice did not enter the cells (non-progeric or progeric) without lipofectamine (Fig. 3C) under the experimental conditions described in Fig. 2A. These data suggested that the impact of progerin on tau aggregation is mediated downstream of seed uptake.

### Progerin expression does not alter the protease resistant core of misfolded tau

**Fig. 4:**
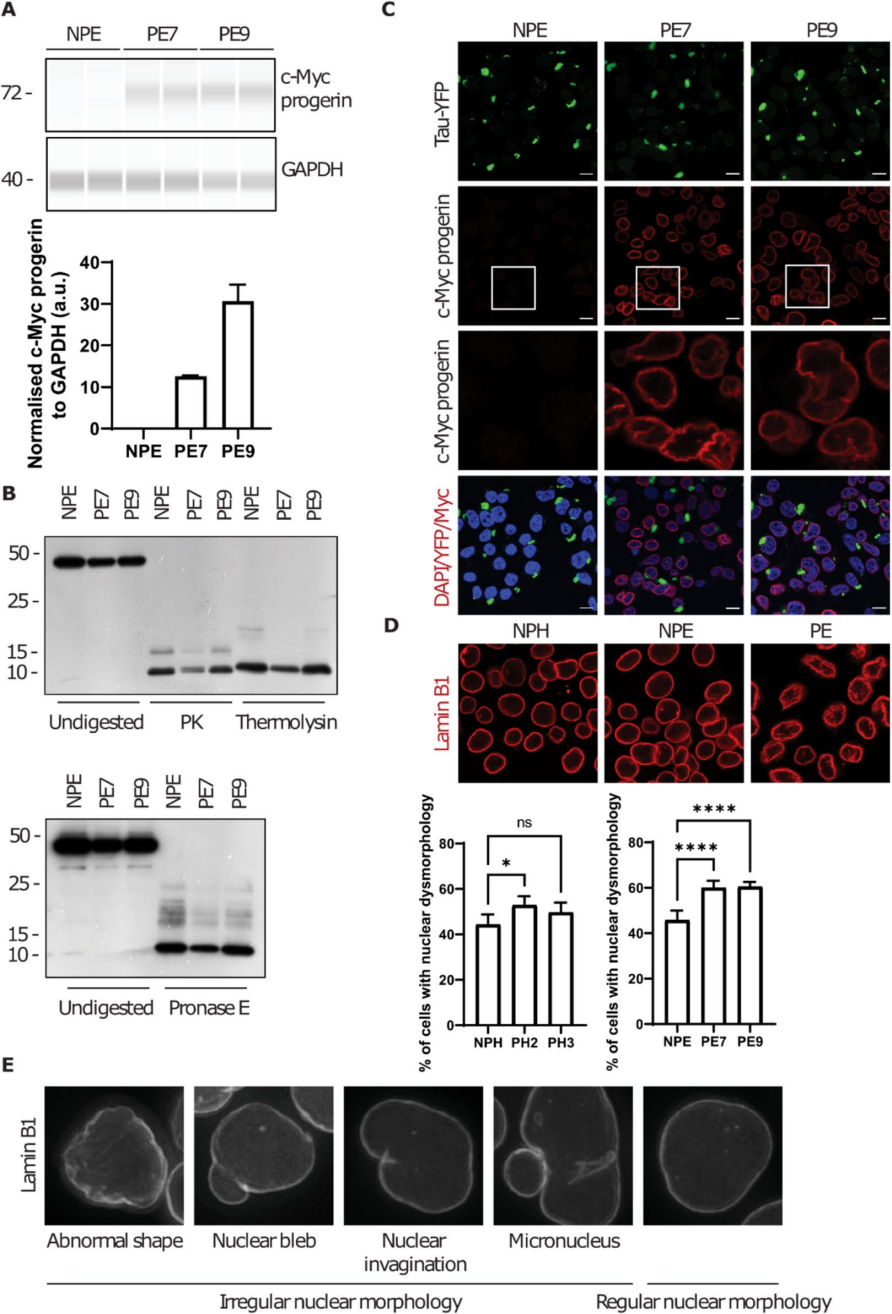
Characterization of progeric cells that are chronically burdened with tau aggregates. **A** Progeric ES1 derivative cells PE7 and PE9 stably express progerin to a similar level. **B** Limited proteolysis (proteinase K, thermolysin and pronase E) demonstrates no changes in the signature of tau aggregates in ES1 cells that stably express progerin. **C** Immunocytochemistry analysis displays nuclear membrane-bound c-Myc-progerin. c-Myc tagged progerin was probed by anti-c-Myc antibody (primary) and Alexa Flour 647 (secondary). Scale bar = 10 µm. **D** Extensive nuclear membrane deformation is observed in progeric ES1 clones in comparison to NPE and NPH cells. Scale bar = 10 µm. One-way ANOVA. *p < 0.05; ****p < 0.0001. **E** Examples of nuclear aberration used for manual annotation in AI-based nuclear morphology analysis

We next addressed the possibility that progerin expression alters a templated misfolding process, the outcome measure being the conformation of the tau moiety in the tau-YFP fusion protein. As different tau conformers associate with different biochemical properties that can dictate their aggregation kinetics [42], enhanced tau seeding efficiency in progeric cells (Fig. 2C) may be a result of misfolded tau adopting a more seeding-prone conformation. To determine if progerin expression associates with changes in a chemical signature of tau aggregates (i.e., as per the fourth hypothesis above), we again turned to the ES1 cell line mentioned above, noting that this was derived from the HEK293 tauRD-YFP reporter cell line by transducing the cells with a preparation of pathogenic tau derived from TgTau^P301L^ Tg mice and assigned a type 2 CSA profile, CSA2 [33]. We then established two progeric derivatives denoted PE7 and PE9; as shown by the capillary western immunoassay, PE7 and PE9 cell lines exhibit similar signals of c-Myc tagged progerin (Fig. 4A). Using the size of a protease-resistant core as a proxy for alternative outcomes of misfolding, three proteases (proteinase K, thermolysin and pronase E) were used to probe the signature of misfolded tau after limited digestion. As the different types of cells (i.e., non-progeric ES1 and progeric ES1 PE7 and PE9) had a protease-resistant core of the same electrophoretic mobility (Fig. 4B), it follows that progerin expression does not alter the conformation of the 10 kDa core of tau aggregates, at least as assessed by this outcome measure.

In the course of these experiments, in contrast to tau inclusion-negative HEK293 cells in which progerin expression only resulted in moderate nuclear deformation (Fig. 1C), progerin expression led to extensive dysmorphology of the nuclear membrane in ES1 cells (Fig. 4C). Using an AI-based nuclear morphology analysis pipeline (Fig. 4E), it became apparent that most progeric ES1 cells exhibited nuclear blebbing (Fig. 4D), this despite steady-state expression of progerin being on average 50% lower in progeric ES1 cells than in progeric HEK derivatives. To this point, quantification of immunoblot data is shown in Figs. 1 and 4 (Fig. 1C and inset, Fig. 4A**)**; setting normalized progerin levels in PH3 cells at 100%, a value of 68% is obtained for PH2 cells and values of 24% and 59% are obtained for PE7 and PE8 cells, respectively. This heightened propensity to nuclear blebbing in the presence of progerin expression and abnormal tau may relate to an intrinsic attribute of the parental ES1 cells, impairment in nucleocytoplasmic transport [40], as discussed below.

### Identification of progerin-regulated proteins in HEK293 tauRD-YFP reporter cells

To begin to uncover the cell biological mechanisms underlying the phenotypic properties of progeric HEK cell lines (including but not limited to elevated tau seeding efficiency), we used a proteomic approach. We performed mass spectrometry-based proteomic analysis to identify proteins with altered expression when progerin is stably expressed in HEK293 tauRD-YFP reporter cell line. Cell lysates were prepared with a urea lysis buffer and proteins from all samples were quantified in a label-free approach after being normalized against the total ion intensity. Compared to the label-based analysis (e.g., tandem mass tag method), a label-free strategy has a higher proteome coverage, comparable reproducibility between technical replicates, but has compromised accuracy in protein quantitation [43]; although some of these challenges have been recently reduced by using data-independent acquisition methods. Over 4000 proteins were collectively detected in the progeric and non-progeric derivatives of HEK cells (Supplementary File 1). These data allowed us to consider three pair-wise comparisons, namely progerin-associated changes versus the parental cell line, tau burden versus the parental cell line and the simultaneous effect of tau burden and progerin expression.

**Fig. 5:**
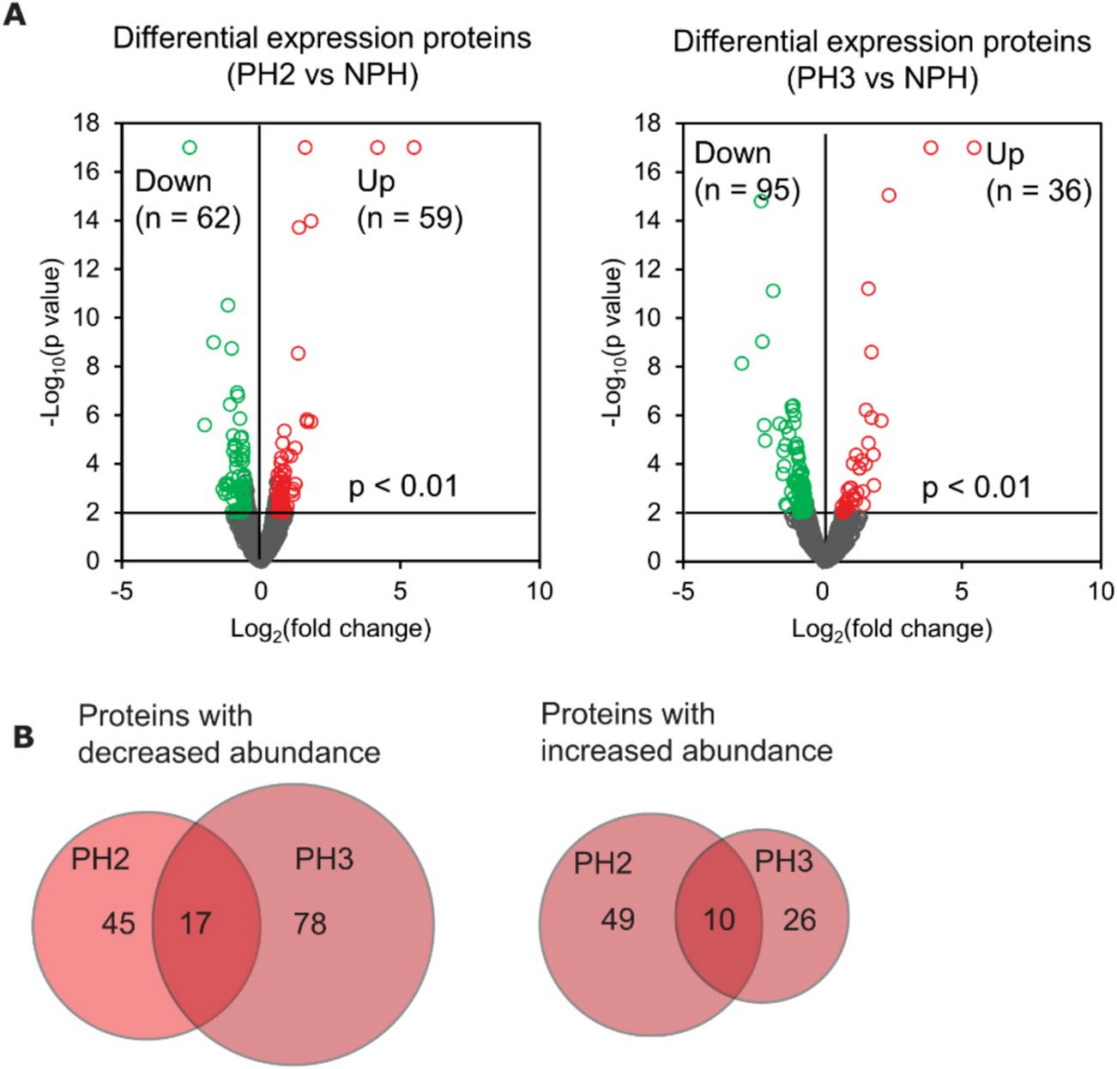
Differential expression analysis of cellular proteome in progeric HEK293 tauRD-YFP cells. **A** Volcano plot displaying the log_2_ fold-change (x-axis) against the − log_10_ statistical *p* value for all proteins differentially expressed between progeric and non-progeric cell lines. Those proteins with significantly decreased expression (*p* < 0.01) are shown in green, while the proteins with significantly increased expression (*p* < 0.01) are noted in red. **B** Venn diagram representation of the number of progerin-specific differentially expressed proteins in HEK293 tauRD-YFP cells. c-Myc tagged progerin is excluded from the diagram

Firstly, we determined the number of proteins differentially expressed in each cell line (PH2 and PH3 compared to NPH). Differential expression proteins (DEPs) were identified as those with > 1.5-fold change in expression level (either increased or decreased between progeric and non-progeric cells) adjusted for a *p*-value of 0.01. Noting lamin A as an internal control for up-regulation, and as demonstrated in the volcano plot in Fig. 5A, we observed 62 less abundant proteins and 59 more abundant proteins in the PH2 cell line and 95 less abundant and 36 more abundant proteins in the PH3 cell line. Among these hits in the proteome, 17 less abundant proteins and 10 more abundant proteins were shared between the two progeric clones PH2 and PH3 (Fig. 5B). Differentially expressed proteins are displayed in a heatmap graphic (Fig. S5).

To obtain unbiased predictions of cellular and biochemical events perturbed in HEK293 tauRD-YFP cells as a consequence of progerin expression, we performed a pathway enrichment analysis using combined lists of differentially expressed protein (140 less abundant and 85 more abundant proteins) present in either PH2 or PH3 cells. Table 1A and B show the top 20 gene ontology (GO) biological processes perturbed in progeric HEK293 tauRD-YFP biosensor cells—the upper panel showing those associated with less abundant proteins and the lower panel showing those associated with more abundant proteins. Apart from pathways related to metabolism and cell cycle regulation, our analysis showed that progeric cells were associated with perturbed protein aggregation-related pathways, including: protein folding (decreased protein level of ARL2, HSPA2, DNAJC7, CCT6B, CHORDC1, TXNDC5); regulation of autophagy (decreased protein level of HMOX1, SLC7A5, CPTP, VDAC1, LRSAM1, and increased protein level of ATM, EEF1A2, CTTN, UCHL1, RB1CC1, CISD1, RPTOR, WNK1), an important pathway for abnormal tau clearance [44]; and positive regulation of proteasomal ubiquitin-dependent protein catabolic process (increased protein level of SUMO2, AURKA, and FBXO22). A significant set of protein hits (17 less and 8 more abundant proteins) are involved in actin cytoskeleton organization, in line with emerging evidence indicating that progerin expression impairs nuclear F-actin and F-actin cytoskeleton stiffness [45, 46]. Additionally, yet contrary to our initial expectations, nuclear chromosome segregation was the only pathway related to perturbation of nucleus organization, with biological processes relevant to nucleocytoplasmic transport, genomic alteration or DNA repair being absent. This may agree, however, with the observation that nuclear membrane irregularities in progeric HEK293 tauRD-YFP cells are minor (Fig. 4D). These data also suggest that the use of zeocin at a relatively low concentration (20 µg/mL) for clone selection did not likely cause DNA damage in the reporter cells.

**Table 1:** Top GO biological processes in progeric HEK293 tauRD-YFP reporter cells

A. Pooled less abundant proteins in progeric HEK293 tauRD-YFP reporter cells ([PH2 vs NPH] U [PH3 vs NPH])			
**GO**	**Biological process**	**Log**_**10**_**(*p*-value)**	**Overlap**
GO:0019752	Carboxylic acid metabolic process	− 10.21	21/783 (2.68%)
GO:0006753	Nucleoside phosphate metabolic process	− 9.83	18/578 (3.11%)
GO:0034655	Nucleobase-containing compound catabolic process	− 7.38	12/333 (3.60%)
GO:0072524	Pyridine-containing compound metabolic process	− 6.86	8/129 (6.20%)
GO:0043648	Dicarboxylic acid metabolic process	− 6.55	7/96 (7.29%)
GO:0016071	mRNA metabolic process	− 5.83	14/646 (2.17%)
GO:0043603	Amide metabolic process	− 4.80	10/403 (2.48%)
GO:0070918	Regulatory ncRNA processing	− 3.88	4/57 (7.02%)
GO:0030036	Actin cytoskeleton organization	− 3.70	10/546 (1.83%)
GO:0006979	Response to oxidative stress	− 3.49	8/375 (2.13%)
GO:0051129	Negative regulation of cellular component organization	− 3.30	11/730 (1.51%)
GO:0006457	Protein folding	− 3.23	6/226 (2.65%)
GO:0019751	Polyol metabolic process	− 3.03	4/95 (4.21%)
GO:0006351	DNA-templated transcription	− 2.43	8/551 (1.45%)
GO:0010632	Regulation of epithelial cell migration	− 2.42	3/69 (4.35%)
GO:0019725	Cellular homeostasis	− 2.32	9/703 (1.28%)
GO:0010507	Negative regulation of autophagy	− 2.03	3/95 (3.16%)
**B. Pooled more abundant proteins in progeric HEK293 tauRD-YFP reporter cells** **([PH2 vs NPH] U [PH3 vs NPH])**			
**GO**	**Biological process**	**Log**_**10**_**(*p*-value)**	**Overlap**
GO:0010506	Regulation of autophagy	− 5.14	8/359 (2.22%)
GO:0010564	Regulation of cell cycle process	− 5.00	11/776 (1.42%)
GO:0048232	Male gamete generation*	− 4.77	10/672 (1.49%)
GO:0001666	Response to hypoxia	− 4.64	7/305 (2.30%)
GO:0098813	Nuclear chromosome segregation	− 4.31	6/234 (2.56%)
GO:0018193	Peptidyl-amino acid modification	− 4.18	7/361 (1.94%)
GO:0006091	Generation of precursor metabolites and energy	− 4.02	7/382 (1.83%)
GO:0072331	Signal transduction by p53 class mediator	− 3.86	4/94 (4.26%)
GO:0030866	Cortical actin cytoskeleton organization	− 3.72	3/40 (7.50%)
GO:0044380	Protein localization to cytoskeleton	− 3.46	3/49 (6.12%)
GO:2,000,573	Positive regulation of DNA biosynthetic process	− 3.36	3/53 (5.66%)
GO:1,903,829	Positive regulation of protein localization	− 3.32	7/499 (1.40%)
GO:0010638	Positive regulation of organelle organization	− 3.32	7/499 (1.40%)
GO:0043467	Regulation of generation of precursor metabolites and energy	− 3.12	4/147 (2.72%)
GO:1,904,375	Regulation of protein localization to cell periphery	− 3.03	4/155 (2.58%)
GO:0008380	RNA splicing	− 3.02	6/407 (1.47%)
GO:0043588	Skin development*	− 2.93	5/283 (1.77%)
GO:0120031	Plasma membrane bounded cell projection assembly	− 2.85	6/439 (1.37%)
GO:0032436	Positive regulation of proteasomal ubiquitin-dependent protein catabolic process	− 2.82	3/81 (3.70%)
GO:0061458	Reproductive system development*	− 2.78	5/308 (1.62%)
**C. Top GO biological processes in both progeric HEK293 tauRD-YFP reporter clones ([PH2 vs NPH] ∩ [PH3 vs NPH])**			
**GO**	**Biological process**	**Log**_**10**_**(*p*-value)**	**Overlap**
GO:0019725	cellular homeostasis	− 3.62	5/703 (0.71%)
GO:0009205	purine ribonucleoside triphosphate metabolic process	− 3.30	3/190 (1.58%)
GO:0051129	negative regulation of cellular component organization	− 2.55	3/348 (0.86%)
GO:0030036	actin cytoskeleton organization	− 2.00	3/546 (0.55%)

Since the phenotype of elevated tau seeding efficiency was observed in both progeric clones (Fig. 2C), we subsequently performed pathway enrichment analysis with the 13.5% of DEPs that were shared by PH2 and PH3 cells (17 less and 10 more abundant proteins versus 225 proteins in the above dataset). The data in Table 1C showed four GO biological processes associated with all significantly modulated proteins that are common in PH2 and PH3 cell lines. The top-ranked functions were associated with cellular homeostasis, purine ribonucleoside triphosphate metabolic process, negative regulation of cellular component organization, and actin cytoskeleton organization.

### Identification of differentially expressed proteins in ES1 versus parental cells

Non-progeric ES1 cells offer a valuable model to investigate generic tau aggregation-related cellular changes for two reasons: (1) ES1 cells are chronically “burdened” with intracellular tau aggregates that do not disappear with passage number; (2) ES1 cells can exhibit all the aforementioned tau inclusions morphologies (amorphous, nuclear envelope, speckles and threads) seen in acute protein transduction of sarkosyl-insoluble tau from mouse or human P301L brain samples [33, 40, 47].

Thus, in a second pairwise comparison, when compared with the parental reporter NPH cell line, our proteomic analyses revealed 49 significantly more abundant proteins and 121 significantly less abundant ones in ES1 cells. Differentially expressed proteins are displayed in a heatmap graphic (Fig. S5). As anticipated, microtubule cytoskeleton organization, regulation of protein polymerization, and supramolecular fiber organization became emphasized in pathway analysis for more abundant proteins (Table S1), highlighting the biological effects of persistent tau aggregate formation in ES1 cells. Proteins with reduced abundance are generally associated with amino acid metabolism, which may relate to the reduced proliferation rate of NPE cells compared to NPH cells. In contrast to initial expectations, none of the identified pathways were related to the functions of the nuclear envelope (e.g., nucleocytoplasmic transport), although defects of the nuclear envelope are present in the tauopathy literature, e.g., nuclear membrane-bound tau condensates [40] and nuclear dysmorphology in FTLD-MAPT neurons [29]. This is, however, in agreement with mild nuclear dysmorphology of non-progeric ES1 cells.

### Identification of progerin-regulated proteins in ES1 cells

Finally, as increased chronological age and tau aggregation are often concurrent variables in human tauopathies, in a third comparison we sought to define the proteomic changes incurred when two stressors, progerin expression and tau aggregation, are present simultaneously in the tau reporter cell derivatives of HEK293 cells. To this end we performed proteomic analysis upon two independently generated progeric ES1 cell lines, PE7 and PE9.

**Fig. 6:**
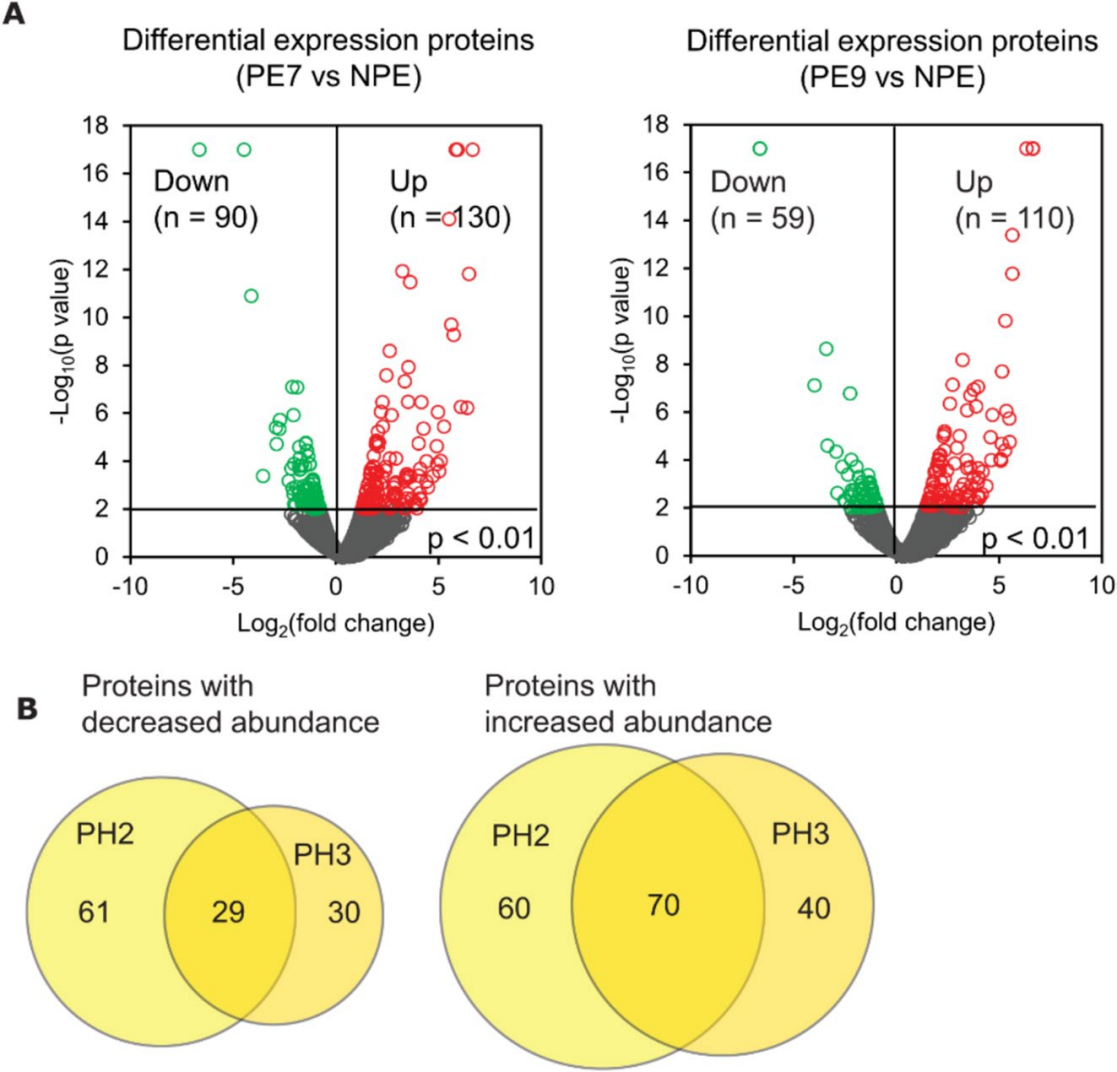
Differential expression analysis of cellular proteome in progeric ES1 cells. **A** Volcano plot displaying the log_2_ fold-change (x-axis) against the − log_10_ statistical *p* value for all proteins differentially expressed between progeric and non-progeric cell lines. Those proteins with significantly decreased expression (*p* < 0.01) are shown in green, while the proteins with significantly increased expression (*p* < 0.01) are noted in red. **B** Venn diagram representation of the number of progerin-specific differentially expressed proteins in ES1 cells. c-Myc tagged progerin is excluded from the diagram

When comparing the progeric ES1 cells to their non-progeric counterpart, we observed 90 less abundant and 130 more abundant proteins in the PE7 cells, and 59 less and 110 more abundant proteins in the PE9 cells (Fig. 6A). Among a total of 149 less abundant and 240 more abundant proteins in the proteome, 29 less abundant proteins and 70 more abundant proteins were identified as overlapping changes in both progeric clones (Fig. 6B). Differentially expressed proteins are displayed in a heatmap graphic (Fig. S5).

GO biological process analysis was also used to determine the pathways contributing to the phenotypic responses observed in progeric ES1 cells. The data in Table 2 showed that abnormalities in the nucleus, including nucleus organization, nucleosome assembly, regulation of protein localization to nucleus, nucleocytoplasmic transport, ribonucleoprotein complex biogenesis, and DNA replication were emphasized in a combined list of differentially expressed protein (90 less abundant and 130 more abundant proteins) in either PE7 or PE9 cells. These changes were not unexpected because progerin expression compromises nuclear integrity and disrupts chromosome organization [48]; and significant nuclear membrane defects were detected in progeric ES1 cells (Fig. 4D). In addition, an upregulation of response to endoplasmic reticulum (ER) stress was strongly emphasized in progeric ES1 cells (Table 2B). Among these proteins, HSPA5, HSP90B1, DNAJC10, DNAJA2, SGTA have chaperone activities. Notably, HSPA5, SGTA, HSP90B1, WFS1, SEC61B, DNAJC10, and STT3B are involved in the ubiquitin-dependent ER-associated degradation (ERAD) pathway, a critical component of the protein quality control system that targets misfolded proteins in the ER for degradation via the ubiquitin–proteasome system (reviewed by [49]). ER stress is closely related to the unfolded protein response (UPR): under stress conditions, the normal function of the ER is compromised, leading to ER stress; in order to restore cellular homeostasis, UPR triggers signaling cascades to enhance the ER’s protein quality control and degradation of misfolded proteins (reviewed by [50]). Since ER stress has substantial implications in tauopathies, such as impairing tau degradation [51] and affecting tau phosphorylation [52], our observation of an elevated level of ER stress in progeric ES1 cells indicates that progerin-induced aging may activate ER stress-UPR signaling pathways and incur cellular damage in the ES1 tauopathy model.

**Table 2:** Top GO biological processes in progeric ES1 cells

A. Pooled less abundant proteins in progeric ES1 cells ([PE7 vs NPE] U [PE9 vs NPE])			
**GO**	**Biological process**	**Log**_**10**_**(*p*-value)**	**Overlap**
GO:0016071	mRNA metabolic process	− 8.21	16/646 (2.48%)
GO:0006412	Translation	− 8.16	13/397 (3.27%)
GO:0022613	Ribonucleoprotein complex biogenesis	− 5.43	11/483 (2.28%)
GO:0034655	Nucleobase-containing compound catabolic process	− 4.25	8/333 (2.40%)
GO:0140694	Membraneless organelle assembly	− 4.08	8/352 (2.27%)
GO:1,901,661	Quinone metabolic process	− 4.05	3/22 (13.6%)
GO:0009060	Aerobic respiration	− 3.28	5/165 (3.03%)
GO:0009408	Response to heat	− 3.28	4/93 (4.30%)
GO:1,903,312	Negative regulation of mRNA metabolic process	− 3.16	4/100 (4.00%)
GO:0006260	DNA replication	− 2.93	5/198 (2.53%)
GO:1,904,951	Positive regulation of establishment of protein localization	− 2.68	6/331 (1.81%)
GO:0044282	Small molecule catabolic process	− 2.53	6/355 (1.69%)
GO:0009451	RNA modification	− 2.33	4/169 (2.37%)
GO:1,903,047	Mitotic cell cycle process	− 2.31	7/523 (1.34%)
GO:0098869	Cellular oxidant detoxification	− 2.23	3/92 (3.26%)
GO:0048193	Golgi vesicle transport	− 2.22	5/289 (1.73%)
GO:0071897	DNA biosynthetic process	− 2.20	3/94 (3.19%)
GO:1,903,432	Regulation of TORC1 signaling	− 2.13	3/100 (3.00%)
GO:0000956	Nuclear-transcribed mRNA catabolic process	− 2.12	3/101 (2.97%)
GO:0016310	Phosphorylation	− 2.11	6/433 (1.39%)
**B. Pooled more abundant proteins in progeric ES1 cells** **([PE7 vs NPE] U [PE9 vs NPE])**			
**GO**	**Biological process**	**Log**_**10**_**(*p*-value)**	**Overlap**
GO:0034976	Response to endoplasmic reticulum stress	− 12.45	16/236 (6.78%)
GO:0045109	Intermediate filament organization	− 10.89	10/75 (13.3%)
GO:0048193	Golgi vesicle transport	− 7.07	12/289 (4.15%)
GO:0051603	Proteolysis involved in protein catabolic process	− 6.69	17/666 (2.55%)
GO:0061024	Membrane organization	− 6.55	18/764 (2.36%)
GO:0033365	Protein localization to organelle	− 6.30	18/794 (2.27%)
GO:0006397	mRNA processing	− 6.15	14/492 (2.85%)
GO:0006997	Nucleus organization	− 5.65	8/152 (5.26%)
GO:0035437	Maintenance of protein localization in endoplasmic reticulum	− 4.34	3/13 (23.1%)
GO:1,903,490	Positive regulation of mitotic cytokinesis	− 4.34	3/13 (23.1%)
GO:0006334	Nucleosome assembly	− 4.24	6/120 (5.00%)
GO:1,905,897	Regulation of response to endoplasmic reticulum stress	− 3.78	5/92 (5.43%)
GO:1,900,180	Regulation of protein localization to nucleus	− 3.77	6/146 (4.11%)
GO:0018149	Peptide cross-linking	− 3.62	3/22 (13.6%)
GO:0030902	Hindbrain development*	− 3.57	6/159 (3.77%)
GO:0043393	Regulation of protein binding	− 3.46	5/108 (4.63%)
GO:0009142	Nucleoside triphosphate biosynthetic process	− 3.44	5/109 (4.59%)
GO:0031398	Positive regulation of protein ubiquitination	− 3.42	5/110 (4.55%)
GO:0006913	Nucleocytoplasmic transport	− 3.30	7/249 (2.81%)
GO:0040007	Growth*	− 3.08	9/441 (2.04%)
**C. Top GO biological processes in both progeric ES1 clones ([PE7 vs NPE] ∩ [PE9 vs NPE])**			
**GO**	**Biological process**	**Log**_**10**_**(*p*-value)**	**Overlap**
GO:0006397	mRNA processing	− 7.17	12/492 (2.44%)
GO:0045109	Intermediate filament organization	− 6.78	6/75 (8.00%)
GO:0002181	Cytoplasmic translation	− 6.60	7/132 (5.30%)
GO:0006334	Nucleosome assembly	− 5.57	6/120 (5.00%)
GO:0010256	Endomembrane system organization	− 4.78	10/571 (1.75%)
GO:0022613	Ribonucleoprotein complex biogenesis	− 4.56	9/483 (1.86%)
GO:0006997	Nucleus organization	− 3.86	5/152 (3.29%)
GO:0140694	Membraneless organelle assembly	− 3.82	7/352 (1.99%)
GO:0032790	Ribosome disassembly	− 3.49	3/41 (7.32%)
GO:0006446	Regulation of translational initiation	− 2.60	3/83 (3.61%)
GO:0030163	Protein catabolic process	− 2.40	8/786 (1.02%)
GO:0031396	Regulation of protein ubiquitination	− 2.38	4/201 (1.99%)
GO:0006979	Response to oxidative stress	− 2.12	5/375 (1.33%)
GO:0001667	Ameboidal-type cell migration	− 2.04	3/132 (2.27%)

To better investigate overlapping changes between two independently generated progeric ES1 cell lines, we used the same analysis pipeline to examine proteomic hits that were shared by these PE7 and PE9 cell lines. As demonstrated by Table 2C, three nuclear processes were altered in progeric ES1 cells: nucleosome assembly, nucleus organization and chromosome localization. These identified pathways further highlighted the toxic effects of progerin on nuclear function in tau inclusion-positive cells. Additionally, progeric ES1 cell were characterized by disruptions of genes involved in protein aggregation-related pathways: regulation of protein ubiquitination and regulation of proteasomal ubiquitin-dependent protein catabolic process, a principal cellular protein degradation pathway responsible for the clearance of misfolded tau [53, 54] and other protein aggregates in neurodegenerative diseases (reviewed by [55–57]). This is in agreement with the activation of ER stress-related pathways shown in Table 2B. Importantly, mRNA processing also emerged as a significantly affected biological process in aging. Several RNA-binding and splicing-associated proteins, including HNRNPC, HNRNPK, ESS2, BUD31, SRSF11, U2AF2, TARDBP, SSU72, LUC7L3, and FIP1L1, were downregulated in progeric ES1 cells. This observation is particularly notable given recent evidence that co-transcriptional splicing is compromised with age [58], further linking impaired RNA processing with the cellular aging phenotype. Overall, the biological processes shown in Table 2 may offer additional explanation to increased tau seeding efficiency observed in progeric HEK cells (PH2 and PH3, Fig. 2).

Top GO biological processes associated with downregulated and upregulated proteins determined by Metascape pathway analysis of progeric HEK293 tauRD-YFP reporter cells. Overlap is defined as the ratio of the number of protein hits from the data set that map to the pathway divided by the total number of molecules within the specified pathway.

*Biological processes that apply only at the level of an organism.

Top GO biological processes associated with downregulated and upregulated proteins determined by Metascape pathway analysis of progeric ES1 cells. Overlap is defined as the ratio of the number of protein hits from the data set that map to the pathway divided by the total number of molecules within the specified pathway.

*Biological processes that apply only at the level of an organism.

## Discussion

### Cellular systems with progerin-based accelerated aging; production and profiling

Cell culture models provide a means to explore causal relationships between genetic mutations, cellular phenotypes, and proteomic alterations, to improve understanding of cascading events in pathogenesis that define neurological outcomes. In the context of aging and neurodegeneration, as discussed previously, LMNA has been reported to play an important role in the central nervous system [59, 60], although there is an ongoing controversy about the regulation of LMNA by a brain-specific microRNA [23]. Progerin, the LMNA-Δ50 form of lamin-A produced by alternative splicing of a mutant allele, is the proximal cause of HGPS pathological aging [16], with the involvement of this spliced form arising from a WT *LMNA* allele confirmed in certain tissues in the context of chronological aging [61]. Due to its potential to circumvent the fixed timeline of chronological aging in a lab setting, progerin has been used to assist in modeling pathological phenotypes of late-onset Parkinson’s disease in human iPSC-derived neurons [12].

By selecting two independent HEK293 tauRD-YFP reporter clones (PH2 and PH3) that stably express progerin, we observed that c-Myc tagged progerin localized at the nuclear envelope, caused minor nuclear dysmorphology in a subpopulation of cells (Figs. 1C and 4D) and did not lead to spontaneous tau aggregate formation (Fig. 1D). As expected, increased levels of double-stranded DNA damage markers were observed in both progeric HEK clones (Fig. 1F and G). After exposing the cells to preparations containing pathogenic tau, both progeric HEK cell lines associated with a higher percentage of seeded population (Fig. 2C). Subsequent experiments (Figs. 3 and 4) demonstrated that progerin expression had no impact on the cellular level of tauRD, transfection efficiency, liposome-independent tau uptake and conformational features of aggregated tau, implying that progeric cells have an increased ability to amplify or accumulate pathogenic tau forms produced by templated misfolding (i.e., as opposed to receiving more tau seeds or altering tau “strain” properties). While there is an unresolved debate as to why lamin A is uniquely regulated by microRNA miR9 in the brain [23], our observations do suggest that miR9-mediated downregulation of *LMNA* may serve as a protection mechanism against the accumulation of pathogenic forms of tau.

An important facet of our study was to evaluate tau seeding events in tau reporter cell lines. Using an automated analysis pipeline (Fig. 2A), the number of tau inclusion-positive cells could be quantified with high accuracy. Future studies may include additional sources of pathogenic tau, such as human AD brain extract and in vitro generated tau fibrils, to investigate how progeric HEK cells respond to different tau seeds. As our observations for progeric HEK cells were generally consistent between PH2 and PH3 clones, the phenotypic changes are likely due to progerin expression, as opposed to spontaneous cell variants unrelated to the biological properties of the introduced expression constructs arising during the subcloning procedure.

To further investigate the molecular mechanisms linking progerin-mediated aging to tau aggregate formation, we conducted mass spectrometry-based proteomic comparison between HEK293 tauRD-YFP cells with/without stable progerin expression. In general, progeric HEK293 tauRD-YFP cells displayed dysregulation of genes associated with cell cycle control and metabolism. Most importantly, pathway analysis on two independently generated progeric cell lines (i.e., PH2 and PH3, biological replicates) revealed perturbed protein aggregation-related pathways in progeric cells (Table 1), which may contribute to increased tau seeding efficiency in these cell lines. Next, to assess whether or not there are synergistic effects of progerin expression and intracellular tau inclusions, we conducted the same analysis with ES1 cells with/without stable progerin expression. In agreement with the observation of overt changes in nuclear morphology in progeric ES1 cells (Fig. 4D), disrupted nuclear functions were revealed by proteomic analysis (Table 2). Additionally, progeric ES1 cells were characterized by disturbed regulation of protein ubiquitination and elevated levels of ER stress (Table 2), suggesting that the protein degradation mechanisms in cells are compromised when tau aggregates and progerin are simultaneously present.

### Summary and perspective

Using progeric HEK293 tauRD-YFP and progeric ES1 cell lines, we demonstrated that HGPS aging was associated with alterations in (i) cellular phenotype, (ii) tau aggregation parameters, and (iii) proteomic analyses that are related to tau biology. These initial observations in a cell culture model indicate that stable expression of progerin as a genetic manipulation may recapitulate or emphasize mechanistic relationships between aging and tau misfolding. In the future, the use of cells that express the full-length tau construct (as opposed to the K18 fragment used in this study) and expansion to animal models of tauopathy may offer insights into the seeding properties dictated by full-length 3R and 4R spliced forms of *MAPT* mRNAs. In the context of cellular aging and protein misfolding, the molecular linkages between nuclear envelope proteins and protein aggregates may warrant further investigation. For instance, oligomeric tau binds to lamin proteins and disrupts the nuclear envelope [62]; inactivation of a nuclear envelope protein, BANF1 (Barrier to autointegration factor 1), leads to an increased abundance of insoluble tau in a cell culture model [63]; mutated BANF1 (A12T) has aberrant interactions with lamin A/C in Nestor-Guillermo progeria syndrome [64], noting that the findings here suggest that disrupted lamin A function aggravates tau aggregation in reporter cells. In addition, progeric tau reporter cell lines with/without intracellular tau aggregates could be integrated into a phenotype-based drug discovery pipeline in an attempt to identify compounds that counteract tau aggregation or cellular aging.

## Supplementary Information

Below is the link to the electronic supplementary material.

ESM 1 (DOCX 6.15 MB)

ESM 2 (XLSX 2.24 MB)

## Data Availability

The data presented in this study are available upon request to the corresponding author.
