# Supplementary material for: Expression of progerin enhances disease-related endpoints in a tau seeding reporter cell system
Source: GeroScience. 2025 Jul 15;48(2):2365–87. doi: 10.1007/s11357-025-01737-z (PMC12972399; doi:10.1007/s11357-025-01737-z)
Supplement: Supplementary file 1 — (DOCX 6.15 MB) [file 11357_2025_1737_MOESM1_ESM.docx]

**Journal: GeroScience**

**Expression of progerin enhances disease-related endpoints in a tau seeding reporter cell system**

Zhuang Zhuang Han^1,2 *^, Sang-Gyun Kang^2^, Erik Gomez-Cardona^1^, Serene Wohlgemuth^2^, Klinton Shmeit^2^, Luis Arce^1,2^, Jiri G. Safar^3^, Olivier Julien^1^, David Westaway^1,2^

^1^ Department of Biochemistry, University of Alberta, 474 Medical Sciences Building, Edmonton, AB, T6G 2H7, Canada

^2^ Centre for Prions and Protein Folding Diseases, University of Alberta, 204 Brain and Aging Research Building, Edmonton, AB, T6G 2M8, Canada

^3^ Department of Pathology, Case Western Reserve University, Institute of Pathology Building, Rm 406, 2085 Adelbert Road, Cleveland, OH 44106-4907, USA

* Current address: Cambridge Institute for Medical Research, Keith Peters Building, Biomedical Campus, Hills Rd, Cambridge CB2 0XY, United Kingdom

Corresponding author: David Westaway (david.westaway@ualberta.ca)

Table S1: Top GO biological processes in non-progeric ES1 cells

| **Less abundant proteins in non-progeric ES1 cells**  **(NPE vs NPH)** | | | |
| --- | --- | --- | --- |
| **GO** | **Biological process** | **Log_10_(P-value)** | **Overlap** |
| GO:0019752 | carboxylic acid metabolic process | -14.14 | 24/783 (3.07%) |
| GO:0008652 | amino acid biosynthetic process | -12.64 | 10/70 (14.29%) |
| GO:1904851 | positive regulation of establishment of protein localization to telomere | -8.08 | 4/7 (57.14%) |
| GO:0046653 | tetrahydrofolate metabolic process | -6.15 | 4/18 (22.22%) |
| GO:0043648 | dicarboxylic acid metabolic process | -5.61 | 6/96 (6.25%) |
| GO:0055086 | nucleobase-containing small molecule metabolic process | -5.23 | 12/605 (1.98%) |
| GO:1902475 | L-alpha-amino acid transmembrane transport | -5.01 | 5/71 (7.04%) |
| GO:0031667 | response to nutrient levels | -4.40 | 10/510 (1.96%) |
| GO:0072524 | pyridine-containing compound metabolic process | -3.76 | 5/129 (3.88%) |
| GO:1901699 | cellular response to nitrogen compound | -3.63 | 10/633 (1.58%) |
| GO:0051701 | biological process involved in interaction with host | -3.44 | 5/151 (3.31%) |
| GO:0019725 | cellular homeostasis | -3.27 | 10/703 (1.42%) |
| GO:0009066 | aspartate family amino acid metabolic process | -3.23 | 3/41 (7.32%) |
| GO:0043009 | chordate embryonic development * | -2.87 | 9/658 (1.37%) |
| GO:0097237 | cellular response to toxic substance | -2.75 | 4/128 (3.13%) |
| GO:0051017 | actin filament bundle assembly | -2.68 | 3/63 (4.76%) |
| GO:0051289 | protein homotetramerization | -2.63 | 3/66 (4.55%) |
| GO:0034976 | response to endoplasmic reticulum stress | -2.58 | 5/236 (2.12%) |
| GO:1901615 | organic hydroxy compound metabolic process | -2.55 | 7/468 (1.50%) |
| GO:0018193 | peptidyl-amino acid modification | -2.47 | 6/361 (1.66%) |
|  | | | |
| **More abundant proteins in non-progeric ES1 cells**  **(NPE vs NPH)** | | | |
| **GO** | **Biological process** | **Log_10_(P-value)** | **Overlap** |
| GO:0000226 | microtubule cytoskeleton organization | -5.49 | 8/578 (1.38%) |
| GO:0032271 | regulation of protein polymerization | -4.73 | 5/206 (2.43%) |
| GO:0097435 | supramolecular fiber organization | -4.34 | 7/608 (1.15%) |
| GO:0045087 | innate immune response * | -2.82 | 6/791 (0.76%) |
| GO:0030036 | actin cytoskeleton organization | -2.77 | 5/546 (0.92%) |
| GO:0002685 | regulation of leukocyte migration | -2.20 | 3/236 (1.27%) |
| Top GO biological processes associated with downregulated and upregulated proteins determined by Metascape pathway analysis of non-progeric ES1 cells. Overlap is defined as the ratio of the number of protein hits from the data set that map to the pathway divided by the total number of molecules within the specified pathway. Asterisk: biological processes that apply only at the level of an organism. | | | |


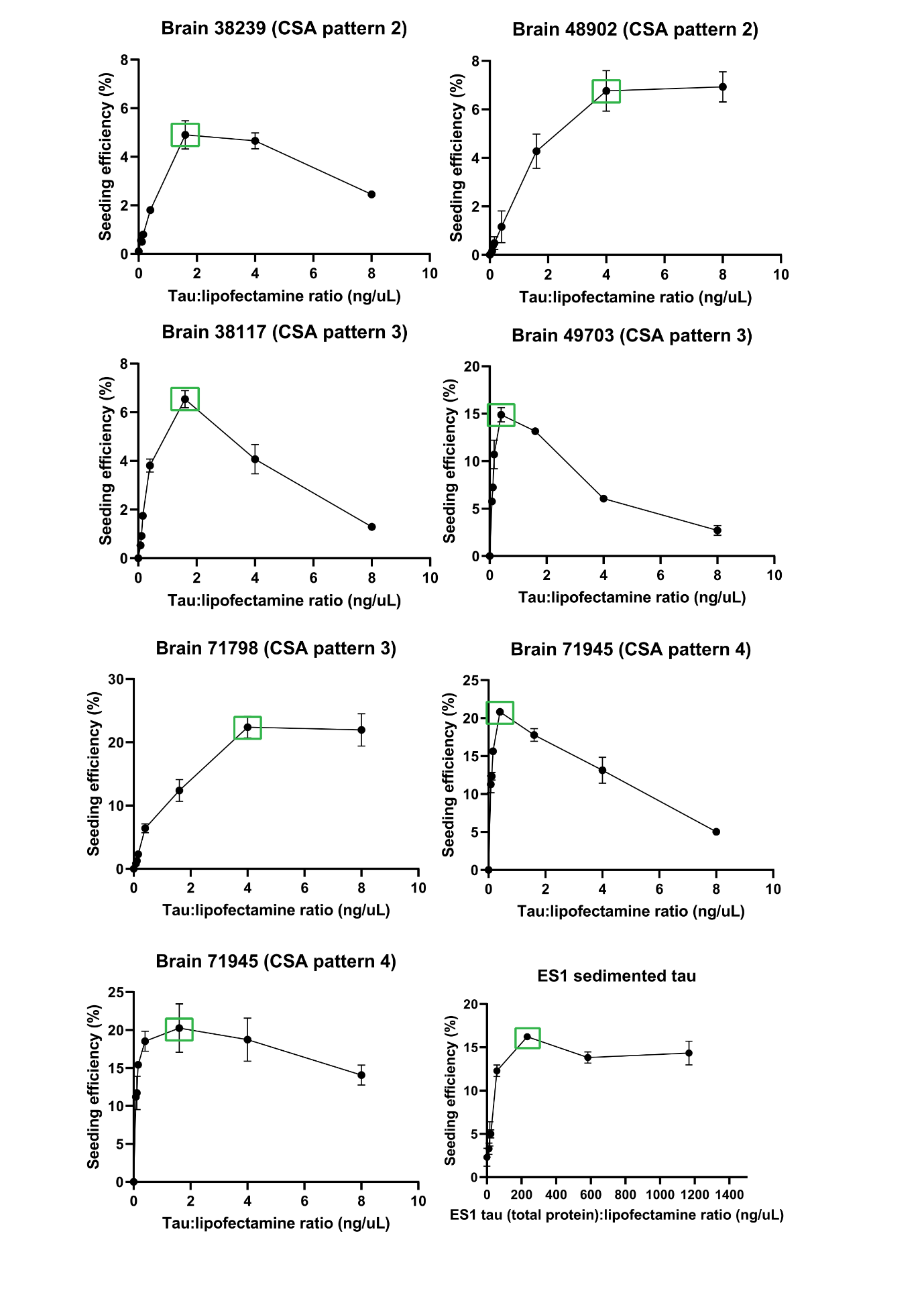


Figure S1: Tau-liposome titration assay used to determine the working ratio between tau extracts (from Tg mouse brain extracts or ES1 cell lysate) and lipofectamine 3000. Optimized tau:liposome ratios (highlighted in green boxes) are determined by 1) the highest seeding efficiency, or 2) the least amount of tau extract with reasonable high seeding efficiency. Error bar = standard error.


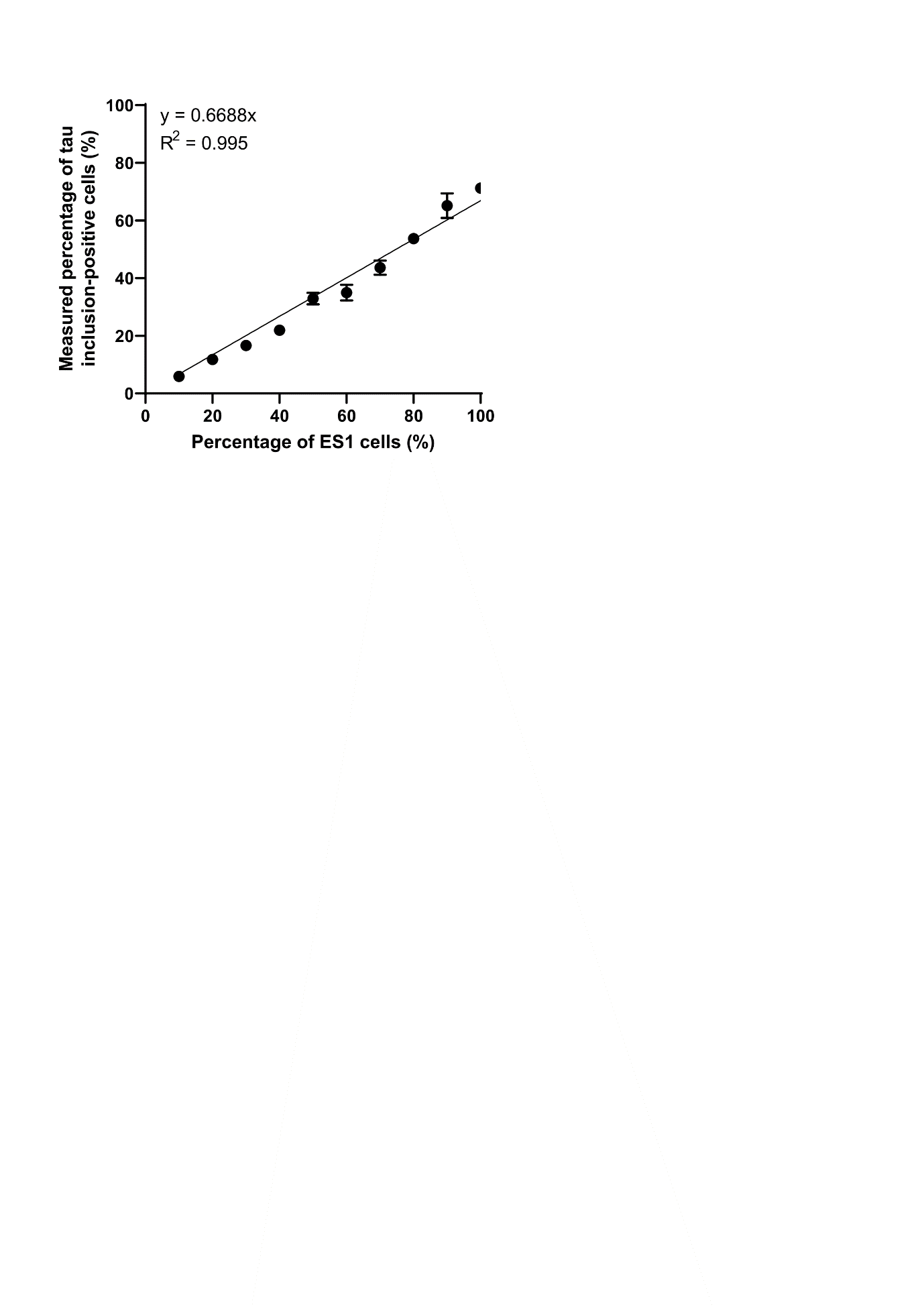


Figure S2: Calibration curve of automated detection of tau inclusion-positive cells. Known percentage of tau-inclusion positive cells (ES1 cells) was plotted on the X-axis and measured percentage of tau-inclusion positive cells was plotted on the Y-axis.


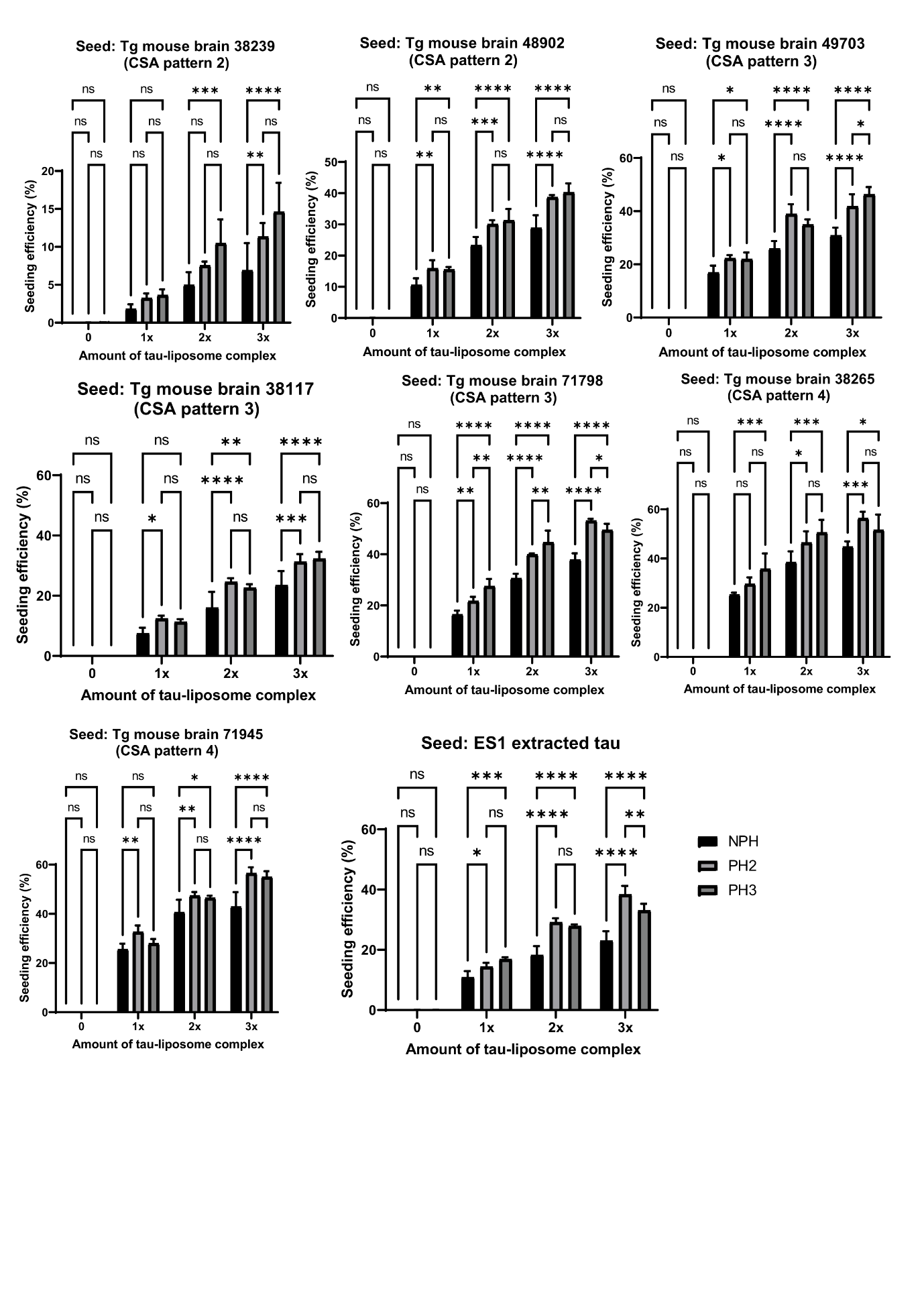


Figure S3: Quantitation of tau seeding efficiency. HEK293 cells (progeric and non-progeric) were transduced with preparations that contain pathogenic tau, including Tg mouse brain extracts and ES1 cell lysate. The seeding efficiency of two progeric clones (PH2 and PH3) is compared to that of non-progeric cells (NPH). Two-way ANOVA; *p<0.05, **p<0.01, ***p<0.001.


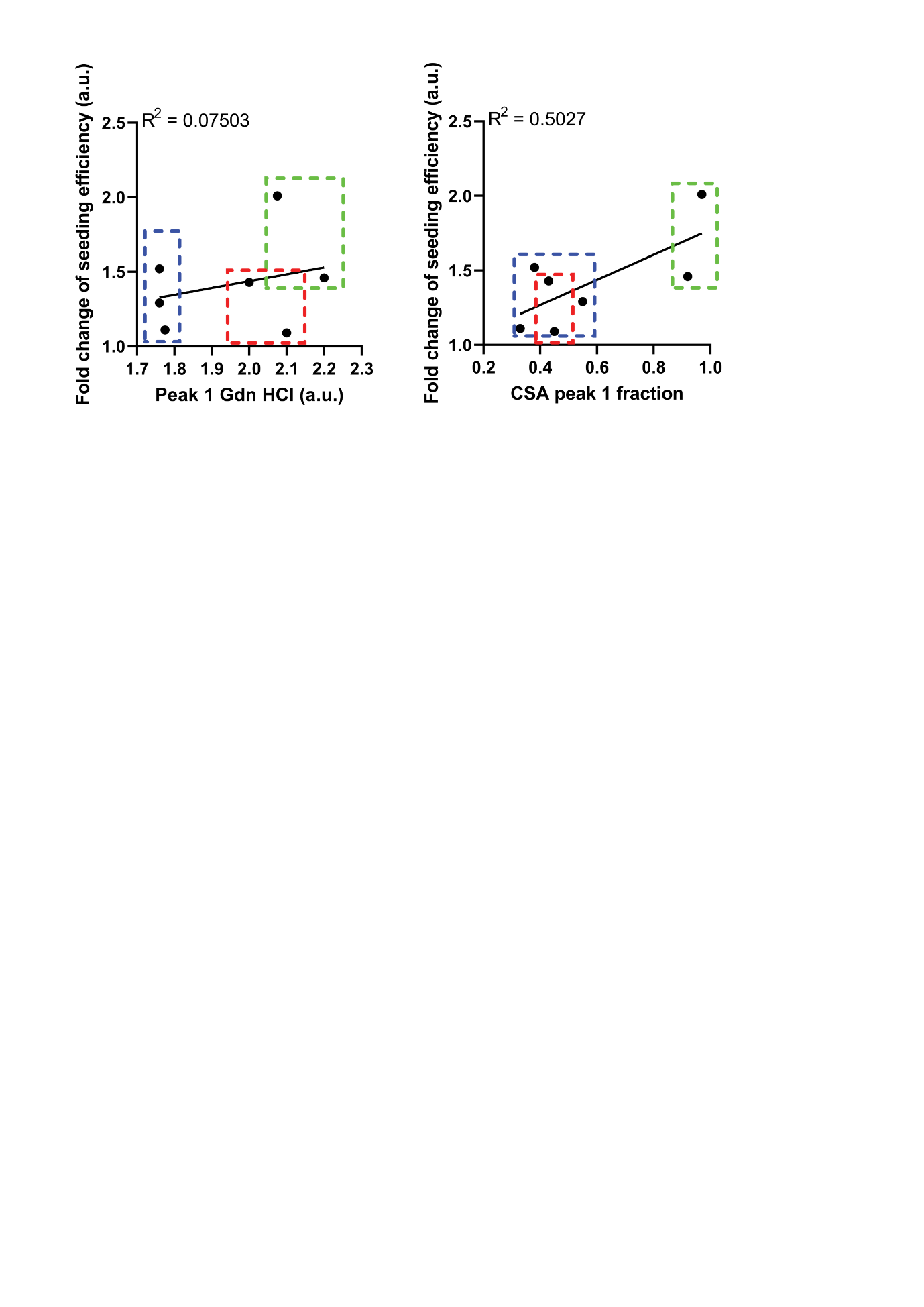


Figure S4: Pearson correlation analysis for progerin-induced increase of seeding efficiency in PH3 cell line and the chemical signature (peak 1 Gdn HCl and CSA peak 1 fraction) generated by the CSA assay. Seven Tg mouse brain extracts were used in the analysis (two brains with CSA pattern 2, three brains with CSA pattern 3 and two brains with CSA pattern 4).


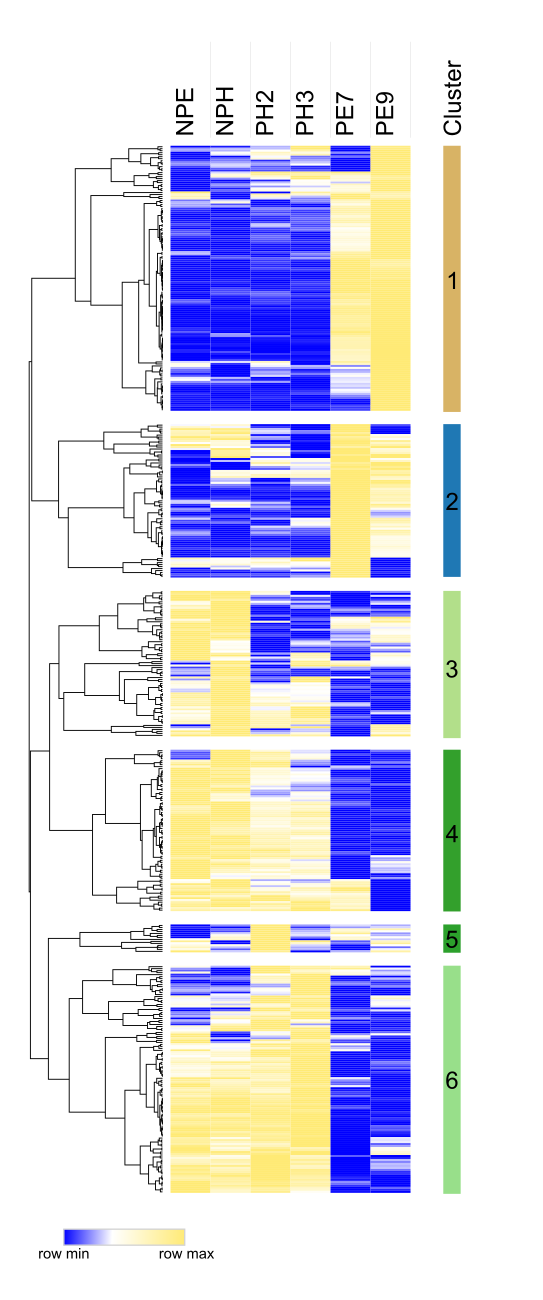


Figure S5: Heatmap of proteins identified in NPE, NPH, PH2, PH3, PE7 and PE9 cells, made with Morpheus. Proteins were grouped based on their abundance patterns among cell types using hierarchical clustering. The hierarchical clustering of these significantly enriched proteins categorizes them based on similar abundance patterns. Proteins with increased abundance in the PE7 and PE9 populations are included in clusters 1 and 2. Proteins with higher levels in the PH2 and PH3 populations are distributed among clusters 5 and 6. The information about the specific proteins included in each cluster can be found at the end of Supplementary File 1.
